# Estimation of Flat Object Deformation Using RGB-D Sensor for Robot Reproduction

**DOI:** 10.3390/s21010105

**Published:** 2020-12-26

**Authors:** Xin He, Takafumi Matsumaru

**Affiliations:** Graduate School of Information, Production and Systems (IPS), Waseda University, Kitakyushu 808-0135, Japan; matsumaru@waseda.jp

**Keywords:** deformation understanding, depth camera, bending line and folding angle, SIFT descriptor, weighted graph clustering, non-rigid point matching, plane detection, region growing, overgrowing avoiding, sliding checking model, deformation reproducing

## Abstract

This paper introduces a system that can estimate the deformation process of a deformed flat object (folded plane) and generate the input data for a robot with human-like dexterous hands and fingers to reproduce the same deformation of another similar object. The system is based on processing RGB data and depth data with three core techniques: a weighted graph clustering method for non-rigid point matching and clustering; a refined region growing method for plane detection on depth data based on an offset error defined by ourselves; and a novel sliding checking model to check the bending line and adjacent relationship between each pair of planes. Through some evaluation experiments, we show the improvement of the core techniques to conventional studies. By applying our approach to different deformed papers, the performance of the entire system is confirmed to have around 1.59 degrees of average angular error, which is similar to the smallest angular discrimination of human eyes. As a result, for the deformation of the flat object caused by folding, if our system can get at least one feature point cluster on each plane, it can get spatial information of each bending line and each plane with acceptable accuracy. The subject of this paper is a folded plane, but we will develop it into a robotic reproduction of general object deformation.

## 1. Introduction

This paper introduces a system that can estimate the deformation process of a deformed flat object (folded plane) and generate the input data for a robot with human-like dexterous hands and fingers to reproduce the same deformation of another similar object. The surface deformation in our system is caused by folding, which means each segmental element of the deformed surface can be regarded as an approximate plane. This paper proposes three core techniques and verifies their effectiveness. By applying these three core techniques to RGB data and depth data, our system can estimate the equations of each bending line, the equations of each plane, and the range of each plane with acceptable accuracy in the camera coordinate system. Based on the spatial relations between the estimated planes derived from the correspondence between two RGB images (an image before transformation and an image after deformation), our system can provide the stepwise deformation process from the initial state to the deformed state. Therefore, our system can generate the input data for a robot with human-like dexterous hands and fingers to perform the same deformation in the future.

Techniques proposed in this paper are improved from conventional studies, and those will contribute as follows:

(1) The first proposal is based on a weighted graph for non-rigid point matching and clustering to find potential planes and establish the correspondence between each pair of planes on two RGB images. Compared to previous methods, it can preserve the neighborhood structure and has better robustness under changing conditions, where an object is deformed with various folding angles or in a complex environment such as with occlusions.
(2) The second proposal uses a refined region growing method for the plane detection which is based on an offset error defined by ourselves. Compared to previous methods, it can detect a 3D plane from depth data and avoid overgrowing without increasing the computational load, thereby improving accuracy.
(3) The third proposal is the development of a novel sliding checking model which consists of four three-dimensional (3D) points and three line segments. The checking model can confirm the adjacent relationship between each pair of planes. Therefore, it is possible to estimate the deformation process based on the obtained information and reproduce the same deformation.

This paper’s remainder is organized as follows: Section 2 introduces the related works and previous studies about dealing with the deformed object, which include the deformable surface 3D reconstruction, non-rigid point matching, and plane detection on depth data. Section 3 explains details of the proposed methods, including the weighted graph clustering method, the refined region growing plane detection method, and the sliding checking model. Section 4 presents several experiments to confirm the effectiveness of proposed techniques and implement the entire system on different deformed papers to check overall performance. Then, we discuss the effective conditions and introduce a way to generate input data for a robot to reproduce the same deformation in the future. Section 5 summarizes the paper and discusses the future works.

## 2. Previous Works and Related Studies

### 2.1. Deformable Surface 3D Reconstruction

About dealing with the deformable surface or object, one of the most popular research works is the 3D reconstruction of the deformable surface with a single camera. According to the different data source, the related studies can be mainly divided into two types: (1) RGB image only; (2) Data from RGB-D cameras.

#### 2.1.1. RGB Image Only

The main challenge of reconstruction of a deformable surface from monocular RGB images is the ambiguity that some different shapes can have a very similar 2D projection on RGB images. For solving the problem, there are two main solutions: (1) Rely on the priori knowledge of the deformable surface, (2) Non-rigid structure-from-motion (NRSFM) techniques.

(1) The priori knowledge can be a model or a reference image with point correspondence. The earliest models are physics-based models, Cohen et al. [1] proposed a 3D generalization of a balloon model as a 3D deformable surface to get greater stability and faster convergence. Metaxas et al. [2] developed a physics-based dynamic model that can incorporate the mechanical principles of rigid and non-rigid bodies into conventional geometric primitives.

Because the physics-based models sometimes are necessary to design very complex objective functions and require the knowledge of material properties of the target surfaces, some deformation models from training data are raised in [3,4,5]. However, the disadvantage of the models from training data are that we need a lot of data for training, and, for different objects, we need different data to train a specific model, so this approach is inefficient for various shapes or different objects. In other directions, Malti et al. [6] proposed the first pixel-based method for 3D reconstruction of a deformable surface from one single view.

(2) The methods that don’t require much priori knowledge are the non-rigid structure-from-motion (NRSFM) methods [7,8,9], and they rely on tracking points over image sequences to recover the deformation shapes as a linear combination of basis shapes. However, linear models can’t reconstruct strongly deforming surfaces composed of multiple local deformations. For solving the limitation of linear models, Gotardo et al. [10] use the kernel trick to model deformable 3D shapes as the output of nonlinear mapping.

Another way to solve the modeling problem of highly deformable surfaces is based on a piecewise model that divides the points to be reconstructed into overlapping areas, and each area is modeled independently [11,12]. This approach can effectively reconstruct highly deformable surfaces if they can be divided into a set of overlapping patches properly. To divide the surface more effectively, Russell et al. [13] proposed a novel energy minimization framework that fits overlapping models.

In order to remove the influence of unwarranted smoothness constraints, Salzmann et al. [14] formulated the reconstruction problem as a Second Order Cone Programming feasibility problem, which can reconstruct highly deformed surfaces. Wang et al. [15] formulated the reconstruction problem as a sequence of Linear Programming (LP) problems and achieved a good performance.

With the huge development of deep learning technology, Tsoli and Argyros [16] proposed a deep learning method for reconstructing a textureless deformable 3D surface from a single RGB image; Bednarik et al. [17] introduced a general framework to learn to reconstruct texture-less deformable surfaces.

As seen in the above descriptions, some techniques can provide acceptable results. However, in most cases, the result is a continuous global surface in a virtual 3D coordinate, which is hard to be operated in the real world.

#### 2.1.2. Data from RGB-D Cameras

Now, RGB-D cameras such as Microsoft Kinect and Intel RealSense are affordable for most of the users, and these are widely used in the field of non-rigid reconstruction or tracking. The approaches of non-rigid reconstruction by data from RGB-D camera can be classified mainly into two types: (1) Template-based approach; (2) Templateless approach.

(1) Most of the template-based approaches rely on a pre-defined template such as skeleton [18,19], thin plate splines [20] and patch [21]. However, fixed templates can’t solve the case that the topology of the object is dynamically changing, such as fracture, tearing, or cutting. To solve this challenge, Tsoli and Argyros [22] proposed a dynamic topology template to track deformable objects that undergo topological changes; Paulus et al. [23] tried to combine a non-rigid augmented reality method with templates to deal with the topological changing. Zhang et al. [24] presented an autonomous pipeline that can build dynamic parametric models.

(2) For many deformable objects that can’t be completely modeled by pre-defined templates, templateless approaches that incrementally reconstruct the object by tracking motion simultaneously are proposed [25]. The main challenge of templateless approaches is the error accumulation during the non-rigid registration. To solve the non-rigid registration problem, Wang et al. [26] proposed a framework consists of local non-rigid bundle adjustment and global optimization; Slavcheva et al. [27] tackle the problem by level set evolution without explicit correspondence search; Yang et al. [28] proposed a global non-rigid registration method for large non-rigid deformations.

RGB-D data can provide higher accuracy in the reconstruction process. Most of the research only focuses on using RGB-D data to get a global reconstruction result, such as a smoothed point cloud. However, this kind of result is hard to be understood by a robot if it needs to do a task such as folding a paper into a special shape.

The comparative overview Table 1 shows the comparison between the different previous methods and our approach. The reasons for using RGB-D data are that depth data can provide higher accuracy while estimating the shape of objects, and RGB data can provide correspondence information which contributes to analyzing the deformation process. Compared to previous methods, the result of the proposed method is a set of segmental elements with their relationship in real space, which makes it easy for robots to understand and operate in the real world with high accuracy. Using our method, a robot with human-like dexterous hands and fingers can perform the deformation operation in order to realize the same deformed status of the paper.

### 2.2. Important Technique for Understanding the Deformed Object

We will propose new techniques for the non-rigid point matching and the plane detection on depth data as the most crucial technique for research about the deformable object. Therefore, here we introduce the previous works for these critical techniques.

#### 2.2.1. Non-Rigid Point Matching

Non-rigid point matching is to establish reliable point correspondences invoked at the same physical points under non-rigid deformation. There are three main strategies of non-rigid point matching: (1) Based on outlier removal by constraints; (2) Based on iteration between correspondence and transformation estimation; (3) Graph-based.

(1) The strategy based on outlier removal is widely used in many research works [29,30,31], and it contains two steps: (a) establish tentative matchings which contain false matchings based on a similarity constraint of feature points (e.g., SIFT(Scale-Invariant Feature Transform) [32]); (b) remove outliers based on geometric constraints. To deal with the case with a large number of outliers, Ma et al. [33] proposed a vector field consensus algorithm that is robust to the case with 90% outliers.

(2) The second strategy is based on a two-step update process, which iteratively alternates between the point correspondence and the transformation estimation. The iterated closest point (ICP) algorithm [34] is a famous method using this strategy, but it is easy to be trapped into local minima for the dispersion of point correspondence. The robust point matching (RPM) algorithm [35] formulated a task as a mixed linear assignment, which is the least square problem and is optimized by a deterministic annealing framework. However, RPM can’t work well when there are missing or extraneous structures, so Sofka et al. [36] proposed a Co-variance Driven Correspondences (CDC) algorithm which can overcome this problem with effective modeling of uncertainty during the registration process.

(3) Recently, the graph-based method has also become a popular strategy to solve the non-rigid point matching problem. One type builds a graph in which each node is a feature point, and it can remove the outliers according to the local neighborhood structures [37,38]. Another type builds the graph in which each node is corresponding and removes the incorrect correspondences by clustering [39,40]. As the development of the second type, Tarashima et al. [41] model the problem as mining of visual shared patterns, which can find the visually similar parts in an image pair, but they use many fixed thresholds in the spatial constraints which will reduce the robustness.

Our non-rigid point matching method is compared with other previous methods in comparative overview Table 2. The graph-based methods can preserve local neighborhood structures which is necessary for our target. Therefore, the approach proposed by us is an improved graph-based method sourced from Tarashima team’s work [41]. Rather than using fixed thresholds in spatial constraints to build an unweighted graph, the spatial constraints are used to generate the distance between each point on a weighted graph in our approach. Then, we choose a different clustering algorithm, the Markov Cluster Algorithm [42], which is effective to deal with the weighted graph. Our method has higher robustness under changing conditions, where the object is deformed with various folding angles or in a complex environment such as with occlusions.

#### 2.2.2. Plane Detection on Depth Data

With the emergence of depth-camera technology, plane detection from the depth data is widely used in the computer vision and robotics fields. According to the principles, plane detection methods can be mainly divided into three types: (1) Based on Random Sample Consensus (RANSAC); (2) Based on Hough Transform (HT); and (3) Based on Region Growing.

(1)Random Sample Consensus (RANSAC) [43] is an iteratively randomized model fitting process which can effectively detect large planes but over-simplify some complex structures. To solve the problem, some methods combine RANSAC with other techniques such as Normal Coherence Checking (NCC-RANSAC) [44], mean shift [45], and Support Vector Machine (SVM) [46].

(2)3D Hough Transform is used for the plane detection from point cloud data [47]. However, the main drawback of Hough Transform is computational complexity, so various optimized methods have been proposed to speed up the voting process, such as Probabilistic Hough Transform (PHT) [48], Random Hough Transform (RHT) [49], and Kernel-based Hough Transform (KHT) [50].

(3) The methods based on Region Growing exploit the neighboring relationship among points to update the plane iteratively.The Early Region Growing method [51] takes much time to calculate, so some improved methods are proposed, such as the Two-Point-Seed Growing method [52] and Point-Based Region Growing (PBRG) [53]. The distinction of the inliers and the outliers of the current growing plane with high accuracy is important for Region Growing methods. In addition, most of the previous works used different thresholds to identify outliers [54,55,56]. For the thresholds-based Region Growing methods, the overgrowing problem is still a challenge because it is hard to detect the intersection by thresholds. Therefore, Jin et al. [57] proposed a robust depth-driven plane detection (DPD) algorithm and used a two-stage refinement to solve the overgrowing problem.

The comparison between our method and previous studies is shown in comparative overview Table 3. High accuracy and robustness are necessary for our purpose of reproducing the deformation with folding by a robot. However, the time cost also needs to be considered. Therefore, we propose a refined Region Growing method derived from the Jin team’s work [57], which can provide high accuracy with a lower computational load. In the system of [57], a dynamic threshold is used to check whether a pixel of depth image belongs to a plane, and the overgrowing problem is solved by an additional two-stage refinement, which takes too much time. We use a unique error in our approach, called the offset error, which is defined by ourselves to check whether a pixel of depth image belongs to the current growing plane. It can avoid the overgrowing during the iterative growing process without any additional process to reduce a computational load.

## 3. Methodology

**Figure 1:**
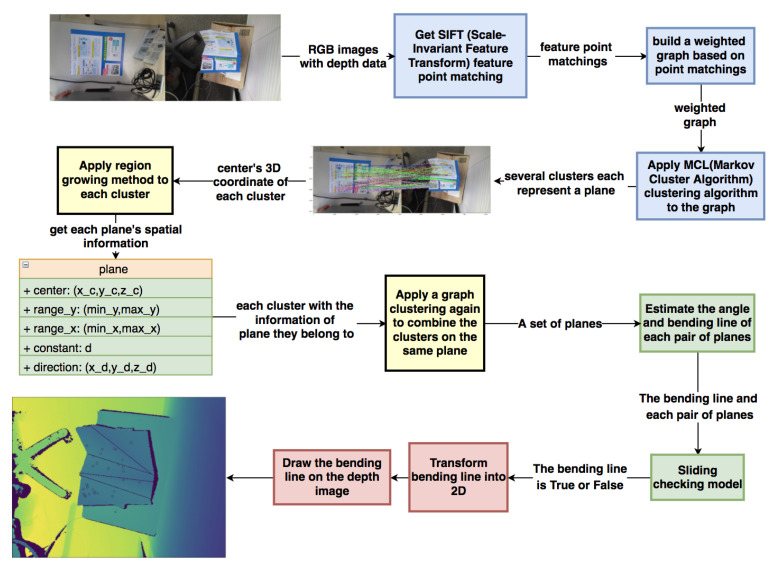
Outline of the proposed method.

The outline of the entire system is shown in Figure 1. Firstly, Microsoft Azure Kinect acquires the RGB image and Depth image of an object both at the initial state and the deformed state. For reproducing the same deformation on an object like paper, it is necessary to find each plane and each bending line on the deformed surface to analyze the task order. The actual procedure to obtain this necessary information with operational accuracy can be divided into three main steps:

(a) The blue blocks of Figure 1 use the weighted graph clustering method to find potential planes and establish the correspondence between each pair of planes on two RGB images. Here, a weighted graph is built based on the dense feature point matchings performed by SIFT (Scale-Invariant Feature Transform) [32] and clustered by the Markov Cluster Algorithm [42] so that the points in each cluster belong to the same plane. The detailed process will be explained in Section 3.1.
(b) After getting a set of clusters, the yellow blocks in Figure 1 use a refined region growing method to estimate each plane and calculate possible bending lines and folding angles between each pair of planes. The details of this part will be explained in Section 3.2.
(c) The green parts of Figure 1 confirm the adjacent relationship between each pair of planes by a novel sliding checking model. There are more details of sliding checking model in Section 3.3. In the red blocks, a projection method is used to check the performance of the entire system, which will be explained in Section 4.3.1.

### 3.1. Non-Rigid Point Matching and Clustering Based on the Weighted Graph

This section is in the blue blocks of Figure 1. Compared with the previous methods, the proposed method can preserve the neighborhood structure and has better robustness under changing conditions. The process of approach can be divided into two parts: (1) Weighted graph construction in Section 3.1.1; (2) Applying Markov Cluster Algorithm to graph in Section 3.1.2.

#### 3.1.1. Weighted Graph Construction

**Figure 2:**
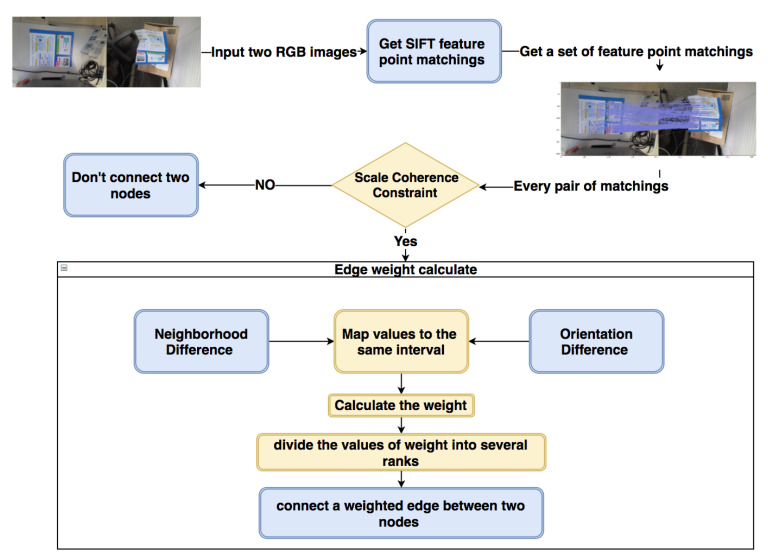
Weighted graph building process.

The outline of the weighted graph construction, which can generate a weighted graph based on the feature point matchings between two RGB images, is shown in Figure 2. The input of this process is two RGB images $Img_{P}$ and $Img_{Q}$. The features of each feature point we need are the scale σ, the orientation θ, and the position *T*.

**Figure 3:**
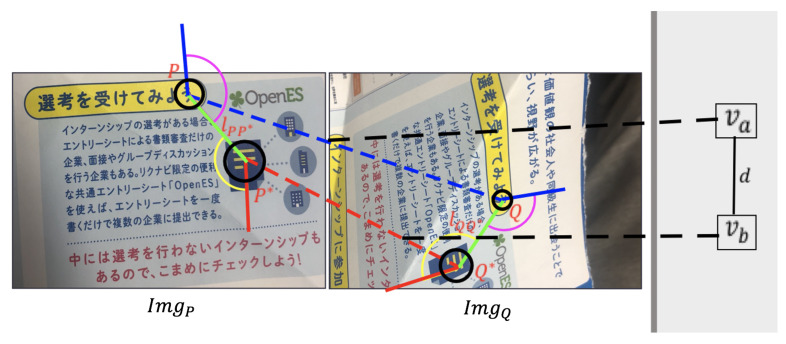
Two pairs of feature point matchings.

An example of two pairs of feature point matchings between $Img_{P}$ and $Img_{Q}$ is shown in Figure 3, where feature points *P* and *Q* constitute a matching $v_{a}=\left(P,Q\right)$ and feature points $P^{*}$ and $Q^{*}$ constitute another matching $v_{b}=\left(P^{*},Q^{*}\right)$. The radii of black circles represent the scales σ of feature points. The blue solid lines represent the orientation $\theta_{P}$ and $\theta_{P^{*}}$, and the red solid lines represent the orientation $\theta_{Q}$ and $\theta_{Q^{*}}$. The green solid line $l_{PP^{*}}$ is the connection between *P* and $P^{*}$, and $l_{QQ^{*}}$ is the connection between *Q* and $Q^{*}$.

The complete process of weighted graph construction is as follows. We firstly use the SIFT [32] descriptor to get the feature point sets $K_{P}$ and $K_{Q}$ from two RGB images $Img_{P}$ and $Img_{Q}$. The reason for choosing the SIFT descriptor to deal with the deformable surface is that it has stable performance in scaling, translation, and rotation. For each SIFT feature point *p*, there are three necessary features that can be easily obtained: the scale $\sigma_{p}\in R$, the orientation $\theta_{p}\in R$, and the position $T_{p}\in R^{2}$. After applying the SIFT feature point matching method, we can get a dense matching result $M_{PQ}$, and the quantity of the matchings is *N*. Each matching $v_{i}=\left(P_{i},Q_{i}\right)$i∈(1,N) in the $M_{PQ}$ represents a node in the weighted graph. After that, the connection relationship and the weight of edge between each pair of nodes can be determined in a new way based on three constraints defined by [41].

For each two feature point matchings $v_{a}=\left(P,Q\right)$ and $v_{b}=\left(P^{*},Q^{*}\right)$ shown in Figure 3, there are three steps to figure out the connection relationship and the weight of edge: (1) Calculate the Scale Coherence; (2) Calculate the Neighborhood Difference and Orientation Difference; and (3) Weight Generation.

**Figure 4:**
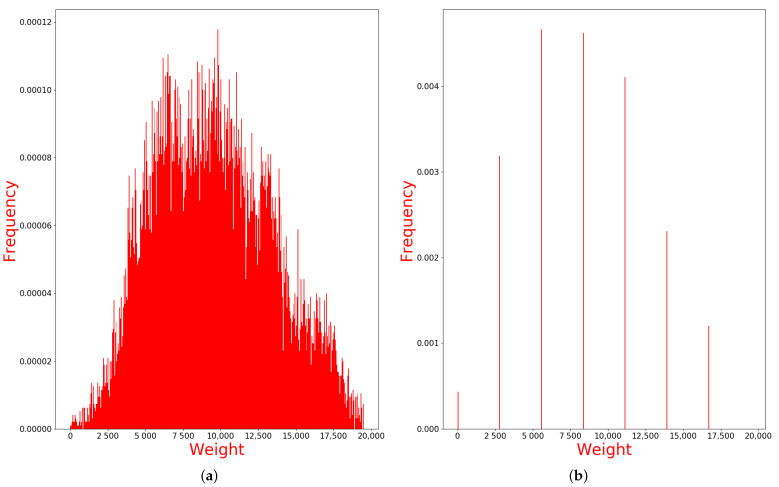
Histogram of weight value (**a**) and Division into seven ranks (**b**).

(1) **Calculate the Scale Coherence**
  For each pair of $v_{a}$ and $v_{b}$, the scale of feature points will be consistent with the scale of the image they belong to, so we use Equation (1) to evaluate the scaling consistency. According to it, some bad matchings that don’t satisfy the scaling consistency can be distinguished and removed. Therefore, if a pair of matchings $v_{a}$ and $v_{b}$ don’t pass the scale coherence checking, we will not use an edge to connect these two nodes:
  (1) $$h_{\sigma }\left(v_{a},\;v_{b}\right)=\left[\frac{1}{\tau_{\sigma }}\leq \frac{\sigma_{P}/l_{PP^{*}}}{\sigma_{Q}/l_{QQ^{*}}}\leq \tau_{\sigma }\right]\wedge \left[\frac{1}{\tau_{\sigma }}\leq \frac{\sigma_{P^{*}}/l_{PP^{*}}}{\sigma_{Q^{*}\prime }/l_{QQ^{*}}}\leq \tau_{\sigma }\right]$$
  In Figure 3, $l_{PP^{*}}$ and $l_{QQ^{*}}$ are the spatial distances of two feature points in each image. With those spatial distances and the scale σ of the feature points in each image, we estimate an error like $\frac{\sigma_{P}/l_{PP^{*}}}{\sigma_{Q}/l_{QQ^{*}}}$ to check the scaling consistency. $\tau_{\sigma }$ is a threshold which is set to $\tau_{\sigma }=1.5$. If $v_{a}$ and $v_{b}$ satisfy the Equation (1) on the scale coherence, there will be an edge between the nodes $v_{a}$ and $v_{b}$ in the weighted graph.
(2) **Calculate the Neighborhood Difference and Orientation Difference**
  The weight of the edge between each pair of nodes will be calculated based on the neighborhood difference and the orientation difference. For each pair of nodes, the more significant the neighborhood difference and orientation difference are, the less likely that they belong to the same plane. Therefore, those two differences and the weight of the edge between the two nodes are positively correlated. Firstly, we need to calculate the neighborhood difference and the orientation difference separately.
  The neighborhood difference is calculated by Equation (2):
  (2) $$d_{neighbor}\left(v_{a},\;v_{b}\right)=\frac{\left(d_{PP^{*}}-min\left(S_{PP^{*}}\right)\right)\times \tau_{N}}{max\left(S_{PP^{*}}\right)-min\left(S_{PP^{*}}\right)}+\frac{\left(d_{QQ^{*}}-min\left(S_{QQ^{*}}\right)\right)\times \tau_{N}}{max(S_{QQ^{*})}-min\left(S_{QQ^{*}}\right)}$$
  where $d_{PP^{*}}$ represents the number of the feature points which are closer to point *P* than point $P^{*}$ in the image $Img_{P}$, and $d_{QQ^{*}}$ represents the number of the feature points which are closer to point *Q* than point $Q^{*}$ in the image $Img_{Q}$. For each pair of *P* and $P^{*}$, we save the value of $d_{PP^{*}}$ in a set $S_{PP^{*}}$; for each pair of *Q* and $Q^{*}$, the value of $d_{QQ^{*}}$ is saved in another set $S_{QQ^{*}}$.
  In Equation (2), to make the $d_{PP^{*}}$ and $d_{QQ^{*}}$ have the same level of influence, we map the $d_{PP^{*}}$ and $d_{QQ^{*}}$ to the same interval $\left(0,\tau_{N}\right)$, where $\tau_{N}$ is the number of possible edges. For example, if the quantity of the weighted graph’s nodes is *N*, then $\tau_{N}=\frac{(N-1)\times N}{2}$.
  The orientation difference is calculated by Equation (3):
  (3) $$O_{\left(v_{a},\;v_{b}\right)}=∥\theta_{P}-∠PP^{*}\left|-\right|\theta_{Q}-∠QQ^{*}∥+∥\theta_{P^{*}}-∠P^{*}P|-|\theta_{Q^{*}}-∠Q^{*}Q∥$$
  In Equation (3), $\theta_{P}$, $\theta_{P^{*}}$, $\theta_{Q}$, and $\theta_{Q^{*}}$ are the orientation of the feature points shown in Figure 3. The $∠PP^{*}$ is the orientation from $T_{P}$ to $T_{P}^{*}$, so $|\theta_{P}-∠PP^{*}|$ represents the pink angle around point *P* in Figure 3, and $|\theta_{P^{*}}-∠P^{*}P|$ represents the yellow angle around the point $P^{*}$ in Figure 3.
  To make the orientation difference have the same level of influence as the neighborhood difference, we also map the orientation difference $O_{\left(v_{a},\;v_{b}\right)}$ to the interval $\left(0,\tau_{N}\right)$ by Equation (4):
  (4) $$d_{orientation}\left(v_{a},\;v_{b}\right)=\frac{\left(O_{\left(v_{a},\;v_{b}\right)}-min\left(L_{\left(v_{a},\;v_{b}\right)}\right)\right)\times \tau_{N}}{max\left(L_{\left(v_{a},\;v_{b}\right)}\right)-min\left(L_{\left(v_{a},\;v_{b}\right)}\right)}$$
  In Equation (4), the $O_{\left(v_{a},\;v_{b}\right)}$ is calculated by Equation (3). For each pair of $v_{a}$ and $v_{b}$, we save the value of $O_{\left(v_{a},\;v_{b}\right)}$ in a set $L_{\left(v_{a},\;v_{b}\right)}$, and the $\tau_{N}$ is the same one in Equation (2).
(3) **Weight Generation.**
  After getting the neighborhood difference and orientation difference, which are mapped to the same interval, we will use them to generate the weight of the edge by Equation (5):
  (5) $$w\left(v_{a},\;v_{b}\right)=d_{orientation}\left(v_{a},\;v_{b}\right)*d_{neighbor}\left(v_{a},\;v_{b}\right)$$
  where we use the product of the neighborhood difference and orientation difference $w(v_{a},\;v_{b})$ to represent the distance between two nodes $v_{a}$ and $v_{b}$. Then, the value of each $w(v_{a},\;v_{b})$ will be saved in a set $M_{\left(v_{a},\;v_{b}\right)}$. With the $M_{\left(v_{a},\;v_{b}\right)}$, we use Equation (6) to map the weight to the interval $\left(0,\tau_{N}\right)$, which is same as the one in Equations (2) and (4):
  (6) $$W\left(v_{a},\;v_{b}\right)=\frac{\left(w\left(v_{a},\;v_{b}\right)-min\left(M_{\left(v_{a},\;v_{b}\right)}\right)\right)\times \tau_{N}}{max(M_{\left(v_{a},\;v_{b}\right)}-min(M_{\left(v_{a},\;v_{b}\right)}}$$
  By Equation (6), we can get the final weight $W(v_{a},\;v_{b})$ of each edge. However, the clustering algorithm will fail while dealing with a large number of continuous values with low discrimination. To avoid the failure of clustering, we need to pre-process the weight $W(v_{a},\;v_{b})$ before applying the clustering algorithm. Therefore, a step is defined in Equation (7) to divide the weight $W(v_{a},\;v_{b})$ into several ranks:
  (7) $$Step=\frac{\tau_{N}}{\delta }$$
  In Equation (7), $\tau_{N}=\frac{(N-1)\times N}{2}$ is equal to the maximum value of $W(v_{a},\;v_{b})$, and δ is the quantity of ranks we want to divide. The value of δ can determine the clustering result by affecting the tightness of the connection in the graph: if δ is too big, a large cluster may be divided into several small clusters; if δ is too small, several different small clusters may be combined into the same cluster. Obviously, too big δ will only increase the subsequent calculation load but will not cause errors, but too small δ can cause worse effects. Through the experiments in different situations similar to Section 4.2.1, we find the δ in the interval (100,200) can almost avoid the situation where the δ is too small. For the case of too large δ, which still may happen, the secondary clustering in Section 3.2.4 can eliminate its influence. Therefore, this assumption can handle most general situations.
  For example, Figure 4a is the histogram of the $W(v_{a},\;v_{b})$ we have gotten. Then, we use the δ=7 to divide the set of the $W(v_{a},\;v_{b})$ into seven ranks, where the ranks will be $\left(0,Step\right),\left(Step,2Step\right),\left(2Step,3Step\right)\cdots \left(6Step,\tau_{N}\right)$. Weight values in each rank will be set to the same value, only the first rank is set to 1, and the rest of the ranks are set to the lower limit of the interval. Then, the weight values of 7 ranks are like (1,1⋯),(Step,Step⋯)⋯(6Step,6Step⋯), which is shown in Figure 4b. However, in the Markov Cluster Algorithm [42], the weight of an edge is the reciprocal of distance, so we take the inverse of ranks to be the final weights. The final weight ranks of the weighted graph are $\left(1,1\cdots \right),\left(\frac{1}{Step},\frac{1}{Step}\cdots \right)\cdots \left(\frac{1}{6Step},\frac{1}{6Step}\right)$. After setting the weight to each edge, the Markov Cluster Algorithm will be applied to get the clusters in the next step.

#### 3.1.2. Applying Markov Cluster Algorithm to the Graph

After getting a weighted graph from Section 3.1.1, we need an effective clustering algorithm to find each cluster from the graph. The reason for choosing the Markov Cluster Algorithm [42] to do the clustering is that it is an effective and efficient method for weighted graph clustering.

**Figure 5:**
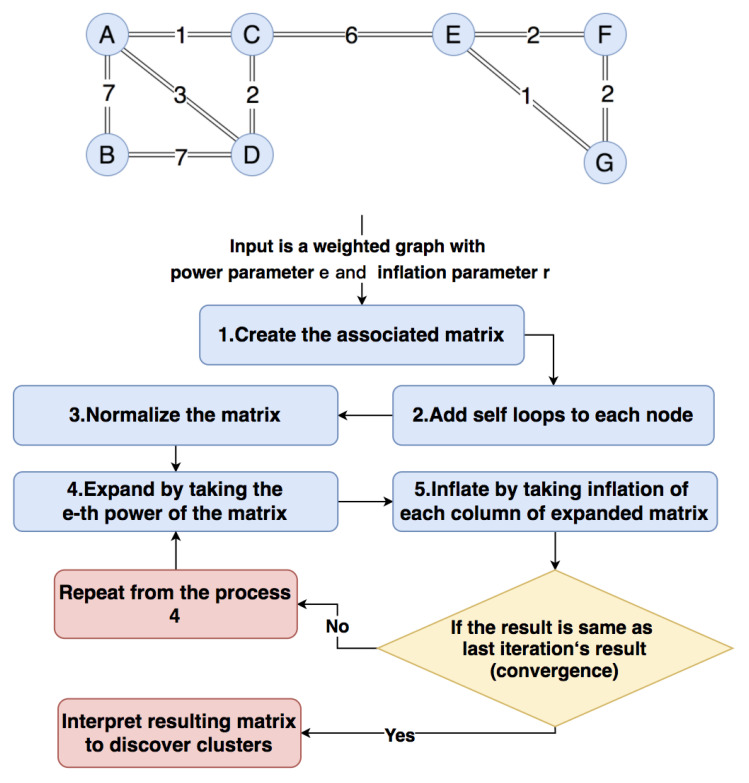
Markov cluster algorithm.

Here, we will introduce the process of the Markov Cluster Algorithm, which is shown in Figure 5. The Markov Cluster Algorithm is based on doing random walks on the graph. If we start from a node and travel to another connected node randomly, the possibility of staying in the current cluster is stronger than moving to another cluster.

The input consists of three parts: (1) A weighted graph from Section 3.1.1; (2) A power parameter *e*; (3) An inflation parameter *r*. Taking the weighted graph in Figure 5 as an example, we construct a 7×7 associated matrix $M_{a}$ (8) in which value $M_{a}\left[i\right]\left[j\right]$ is the weight of the edge between node *i* and node *j*. For example, the distance between *A* and *B* is 7, so the possibility from *A* to *B* is the reciprocal of distance, which is $\frac{1}{7}$. Therefore, the value of $M_{a}\left[A\right]\left[B\right]$ is $\frac{1}{7}$, which is the weight of edge AB calculated in Section 3.1.1.

(8) $$M_{a}=\left[\begin{array}{cccccccc} \; & A & B & C & D & E & F & G\\ A & 0 & \frac{1}{7} & 1 & \frac{1}{3} & 0 & 0 & 0\\ B & \frac{1}{7} & 0 & 0 & \frac{1}{7} & 0 & 0 & 0\\ C & 1 & 0 & 0 & \frac{1}{2} & \frac{1}{6} & 0 & 0\\ D & \frac{1}{3} & \frac{1}{7} & \frac{1}{2} & 0 & 0 & 0 & 0\\ E & 0 & 0 & \frac{1}{6} & 0 & 0 & \frac{1}{2} & 1\\ F & 0 & 0 & 0 & 0 & \frac{1}{2} & 0 & \frac{1}{2}\\ G & 0 & 0 & 0 & 0 & 1 & \frac{1}{2} & 0 \end{array}\right]$$

Then, we should add a self-loop to each node, which means to make $M_{a}\left[i\right]\left[i\right]=1$ for each node *i*. The associated matrix will be like matrix (9).

(9) $$M_{a}=\left[\begin{array}{cccccccc} \; & A & B & C & D & E & F & G\\ A & 1 & \frac{1}{7} & 1 & \frac{1}{3} & 0 & 0 & 0\\ B & \frac{1}{7} & 1 & 0 & \frac{1}{7} & 0 & 0 & 0\\ C & 1 & 0 & 1 & \frac{1}{2} & \frac{1}{6} & 0 & 0\\ D & \frac{1}{3} & \frac{1}{7} & \frac{1}{2} & 1 & 0 & 0 & 0\\ E & 0 & 0 & \frac{1}{6} & 0 & 1 & \frac{1}{2} & 1\\ F & 0 & 0 & 0 & 0 & \frac{1}{2} & 1 & \frac{1}{2}\\ G & 0 & 0 & 0 & 0 & 1 & \frac{1}{2} & 1 \end{array}\right]$$

The next step is normalizing the $M_{a}$ column by column to turn the associated matrix $M_{a}$ into a possibility matrix $M_{p}$. The $M_{p}$ is shown like matrix (10), where the sum of each column is 1, and each column represents the possibility of traveling from the current node to other nodes. For instance, the column $M_{p}\left[i\right]\left[A\right]$ saves the possibility from node *A* to other nodes *i*.

(10) $$M_{p}=\left[\begin{array}{cccccccc} \; & A & B & C & D & E & F & G\\ A & 0.404 & 0.111 & 0.375 & 0.169 & 0 & 0 & 0\\ B & 0.058 & 0.778 & 0 & 0.072 & 0 & 0 & 0\\ C & 0.404 & 0 & 0.375 & 0.253 & 0.062 & 0 & 0\\ D & 0.135 & 0.111 & 0.188 & 0.506 & 0 & 0 & 0\\ E & 0 & 0 & 0.06 & 0 & 0.375 & 0.25 & 0.4\\ F & 0 & 0 & 0 & 0 & 0.187 & 0.5 & 0.2\\ G & 0 & 0 & 0 & 0 & 0.375 & 0.25 & 0.4 \end{array}\right]$$

After getting the possibility matrix, the Expand process is applied to do the random walks. We take the *e*-th power of the possibility matrix $M_{ex}$ to replace the former possibility matrix in Equation (11); here, the value of *e* is set to e=2. Each column in $M_{ex}$ represents the possibility of traveling *e* steps from the current node to other nodes, such as the column $M_{ex}\left[i\right]\left[A\right]$ saves the possibility of traveling two steps from node *A* to other nodes *i*:

(11) $$M_{ex}=M_{p}^{2}=\left[\begin{array}{cccccccc} \; & A & B & C & D & E & F & G\\ A & 0.344 & 0.15 & 0.324 & 0.256 & 0.023 & 0 & 0\\ B & 0.078 & 0.619 & 0.035 & 0.103 & 0 & 0 & 0\\ C & 0.349 & 0.073 & 0.343 & 0.291 & 0.047 & 0.016 & 0.025\\ D & 0.205 & 0.158 & 0.216 & 0.334 & 0.012 & 0 & 0\\ E & 0.025 & 0 & 0.047 & 0.016 & 0.341 & 0.319 & 0.36\\ F & 0 & 0 & 0.012 & 0 & 0.239 & 0.347 & 0.255\\ G & 0 & 0 & 0.023 & 0 & 0.337 & 0.319 & 0.36 \end{array}\right]$$

After doing the random walks, the Inflation process is applied to strengthen strong possibility and weaken weak possibility by re-scaling each column of the matrix $M_{ex}$ with power *r* and normalizing the column. Here, the value of *r* is set to r=2, so we square each column and get a new matrix $M_{r}$ (12):

(12) $$M_{r}=\left[\begin{array}{cccccccc} \; & A & B & C & D & E & F & G\\ A & 0.118 & 0.023 & 0.105 & 0.066 & 0.001 & 0 & 0\\ B & 0.006 & 0.384 & 0.001 & 0.011 & 0 & 0 & 0\\ C & 0.122 & 0.005 & 0.118 & 0.085 & 0.002 & 0 & 0.001\\ D & 0.042 & 0.025 & 0.047 & 0.112 & 0 & 0 & 0\\ E & 0.001 & 0 & 0.002 & 0 & 0.117 & 0.102 & 0.13\\ F & 0 & 0 & 0 & 0 & 0.057 & 0.12 & 0.065\\ G & 0 & 0 & 0.001 & 0 & 0.114 & 0.102 & 0.13 \end{array}\right]$$

Then, normalize each column of $M_{r}$ to make the sum of each column equal to 1, the $M_{r}$ will be like (13):

(13) $$M_{r}=\left[\begin{array}{cccccccc} \; & A & B & C & D & E & F & G\\ A & 0.41 & 0.052 & 0.383 & 0.241 & 0.002 & 0 & 0\\ B & 0.021 & 0.879 & 0.005 & 0.039 & 0 & 0 & 0\\ C & 0.422 & 0.012 & 0.431 & 0.31 & 0.008 & 0.001 & 0.002\\ D & 0.145 & 0.057 & 0.17 & 0.409 & 0 & 0 & 0\\ E & 0.002 & 0 & 0.008 & 0.001 & 0.401 & 0.314 & 0.399\\ F & 0 & 0 & 0.001 & 0 & 0.197 & 0.372 & 0.2\\ G & 0 & 0 & 0.002 & 0 & 0.392 & 0.314 & 0.399 \end{array}\right]$$

Comparing each column of $M_{r}$ with each column of $M_{ex}$, it is evident that the strong neighborhood connection becomes stronger and stronger, and the weak neighborhood connection becomes weaker and weaker. Then, we make the $M_{r}$ to be the new possibility matrix $M_{p}$ and repeat the Expand and Inflation processes. The matrix $M_{p}$ will finally converge to a steady-state matrix $M_{output}$ (14) where every non-zero value of a column has the same value.

(14) $$M_{output}=\left[\begin{array}{cccccccc} \; & A & B & C & D & E & F & G\\ A & 0 & 0 & 0 & 0 & 0 & 0 & 0\\ B & 0 & 1 & 0 & 0 & 0 & 0 & 0\\ C & 1 & 0 & 1 & 1 & 0 & 0 & 0\\ D & 0 & 0 & 0 & 0 & 0 & 0 & 0\\ E & 0 & 0 & 0 & 0 & 1 & 1 & 1\\ F & 0 & 0 & 0 & 0 & 0 & 0 & 0\\ G & 0 & 0 & 0 & 0 & 0 & 0 & 0 \end{array}\right]$$

The clustering result can be obtained by interpreting the final matrix $M_{output}$. We need to loop through each column of $M_{output}$; if $M_{output}\left[i\right]\left[j\right]!=0$, it means that nodes *i* and *j* are in the same cluster. In this case, we can get (A,C,D),(B),(E,F,G) three clusters.

By applying the Markov Cluster Algorithm to the weighted graph from Section 3.1.1, we can get several clusters of feature point matchings. Each cluster of feature point matchings can be divided into two corresponding clusters of feature points that belong to the same area of the object’s surface between two RGB images. If the object in one RGB image is in the initial state, we think the corresponding area in another RGB image is also not deformed. In our case, we only need the cluster of feature points $\Theta_{c}$ in the deformed object’s RGB image. Each $\Theta_{c}$ contains a set of feature points that belongs to the same approximate plane on the deformed object’s surface.

To confirm our method’s improvement in robustness, we compared our approach to the unweighted-graph method of [41] in Section 4.2.1.

### 3.2. Plane Detection on Depth Data

After getting a set of clusters and each cluster, $\Theta_{c}$ can represent an approximate plane of the deformed surface in an RGB image. This section, which is in the yellow blocks of Figure 1, explains a refined region growing plane detection algorithm. Compared with the previous region growing methods, the proposed method can avoid overgrowing without increasing the computational load, thereby improving accuracy.

**Figure 6:**
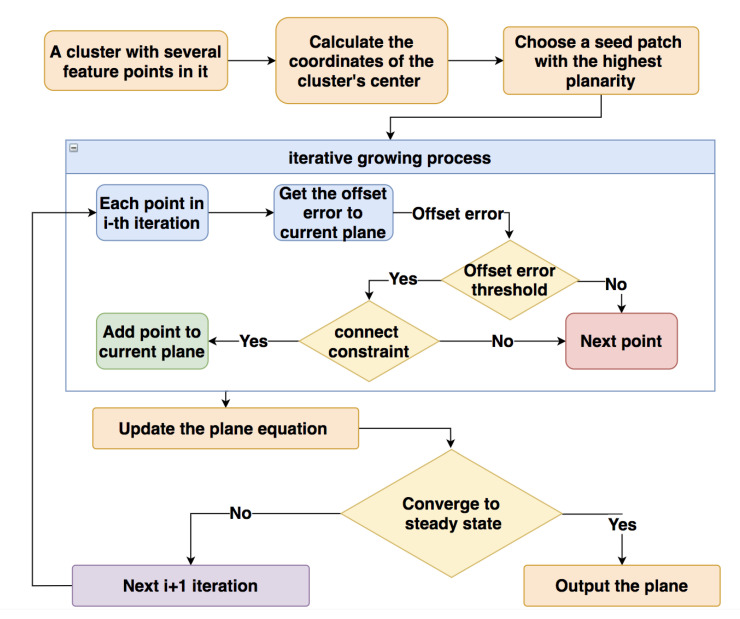
Region growing method.

The complete process of detecting the plane from a cluster $\Theta_{c}$ is shown in Figure 6: firstly, we should find a 7*7 seed patch around the center point of the cluster with the highest planarity. The size of the seed patch should be appropriate: if the size is too small, the seed patch will be more susceptible to local slight deformation in the early growth stage; if the size is too large, the seed patch may be distributed in two planes at the same time, causing huge errors. In order to identify some small flat regions, the size of 7*7 is suitable, but the specific size can be adjusted according to different requirements. An iterative growth process is then applied to extend the current plane from the seed patch for estimating the equation and range of the plane.

#### 3.2.1. Coordinate Conversion

In order to estimate the plane equation, it is necessary to convert the pixels of the depth image to 3D points in the camera coordinate system. The 3D coordinates of each pixel in the camera coordinate system can be obtained with the depth data and the calibration parameters of the camera. Using the self-calibration function provided by Azure Kinect SDK, it is easy to obtain four necessary parameters $f_{x},f_{y},c_{x},c_{y}$. The parameters $c_{x}$ and $c_{y}$ are the 2D coordinate of the image’s center; $f_{x}$ and $f_{y}$ are the proportional parameter to the depth value. For each pixel with position $\left(x_{d},y_{d}\right)$ in image with the depth value dp, the 3D coordinate $\left(x_{w},y_{w},z_{w}\right)$ can be calculate as Equation (15):

(15) $$\begin{array}{cc} x_{w} & =\left(x_{d}-c_{x}\right)\times \frac{dp}{f_{x}}\\ y_{w} & =\left(y_{d}-c_{y}\right)\times \frac{dp}{f_{y}}\\ z_{w} & =dp \end{array}$$

#### 3.2.2. Find a Seed Patch

Before applying the iterative growing process, it is necessary to find a suitable seed block as a starting area. The equation of a plane in 3D space can be represented like ax+by−z+d=0, where the normal vector ñ=(a,b,−1) and d=d is a constant. The plane equation can be estimated by applying a Linear Least Squares plane fitting method to a set of points on a plane. For any 3D point p=(x,y,z), the fitting error between a plane and the point p, which is defined in [57], can be evaluated as:

(16) $$error\left(p\right)=\left|p\cdot ñ+d\right|$$

For a seed patch with a point set P, we calculate the root mean square of fitting error $\phi_{i}$ by Equation (17) to evaluate the planarity of seed patch $S_{i}$, and higher planarity corresponds to smaller $\phi_{i}$:

(17) $$\phi_{i}=\sqrt{\frac{1}{\left|P\right|}\sum_{\forall p\in P}error^{2}\left(p\right)}$$

**Figure 7:**
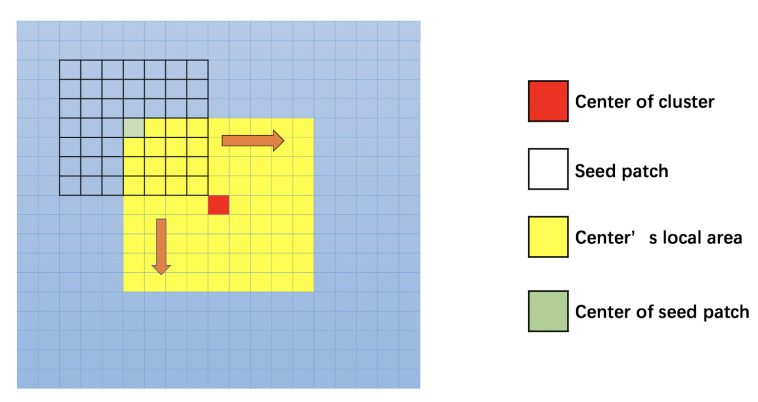
Find the seed patch with the highest planarity.

As the Figure 7 shows, to find a suitable seed patch for the cluster, we take a 9*9 block B, which is centered on the center point of cluster $\Theta_{c}$; each pixel in B is a candidate for the center of seed patch S. The size of B should consider both effectiveness and calculation cost: if the size is too small, the search area will be too small to find the most suitable seed patch; if the size is too large, it will generate excessive calculation load and increase time consumption. In our approach, 9*9 is a suitable size to effectively select suitable seeds and limit the calculation load, but the size also can be adjusted according to different conditions. In the 9*9 case, there are 81 candidates for the seed patch, then the $\phi_{i}$ of each seed patch will be calculated, and the one with the smallest value of $\phi_{i}$ will be a suitable seed patch for growing.

#### 3.2.3. Iterative Region Growing Process

After finding a suitable seed patch, we will introduce details of the iterative growth process, which uses the offset error $error_{o}$ to check each pixel and avoid overgrowing. Before starting the iteration process, it is necessary to estimate the seed patch’s initial equation by the Linear Least Squares plane fitting approach.

Firstly, we will introduce the offset error $error_{o}$ of a point in the depth image. For any 3D point $P_{w}$ with coordinate $\left(x_{w},y_{w},z_{w}\right)$ which is on a depth image $img_{d}$, we can use an inverse process of Equation (15) to calculate the 2D position $\left(x_{d}^{\prime },y_{d}^{\prime }\right)$ of $P_{w}$ on the $img_{d}$:

(18) $$\begin{array}{cc} x_{d}^{\prime } & =\frac{f_{x}\times x_{w}}{z_{w}}+c_{x}\\ y_{d}^{\prime } & =\frac{f_{y}\times y_{w}}{z_{w}}+c_{y} \end{array}$$

**Figure 8:**
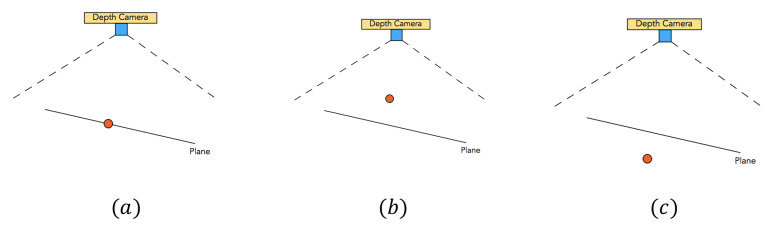
Positional relationship between point and plane: (**a**) on plane; (**b**) above the surface; (**c**) behind the surface.

If $P_{w}$ on the plane like case (a) in Figure 8, the $\left(x_{d}^{\prime },y_{d}^{\prime }\right)$ we calculated is almost equal to the real 2D position $\left(x_{d},y_{d}\right)$ of $P_{w}$ and the depth value $img_{d}\left[int\left(y_{d}^{\prime }\right)\right]\left[int\left(x_{d}^{\prime }\right)\right]=img_{d}\left[y_{d}\right]\left[x_{d}\right]$. If we apply Equation (15) with the real 2D position $\left(x_{d}^{\prime },y_{d}^{\prime }\right)$ and $img_{d}\left[int\left(y_{d}^{\prime }\right)\right]\left[int\left(x_{d}^{\prime }\right)\right]$, we will get a new 3D coordinate $\left(x_{w}^{\prime },y_{w}^{\prime },z_{w}^{\prime }\right)$, which is very close to the $\left(x_{w},y_{w},z_{w}\right)$ only with a tiny rounding error.

If $P_{w}$ is above the plane in the real world like case (b) of Figure 8, $P_{w}$ does not exist in the depth image $img_{d}$. Therefore, the calculated 2D position $\left(x_{d}^{\prime },y_{d}^{\prime }\right)$ represents a point far away from $P_{w}$ in 3D space. If $P_{w}$ is behind the plane in the real world like Figure 8c, it also can’t be caught in the depth image $img_{d}$, and $\left(x_{d}^{\prime },y_{d}^{\prime }\right)$ also represents a far away 3D point.

Both in case (b) and case (c), the 3D point $P_{w}$ is not a point caught in the depth image, so the calculated 2D position $\left(x_{d}^{\prime },y_{d}^{\prime }\right)$ represents a different point, which is caught in the depth image. Because of the multiplication operation in Equation (18), the 3D coordinate $\left(x_{w}^{\prime },y_{w}^{\prime },z_{w}^{\prime }\right)$ of 2D position $\left(x_{d}^{\prime },y_{d}^{\prime }\right)$ will have a big offset to $\left(x_{w},y_{w},z_{w}\right)$.

To evaluate the offset, we use Equation (19) to calculate the offset error $error_{o}$ between two 3D coordinates $\left(x_{w}^{\prime },y_{w}^{\prime },z_{w}^{\prime }\right)$ and $\left(x_{w},y_{w},z_{w}\right)$:

(19) $$error_{o}=\sqrt{{\left(x_{w}^{\prime }-x_{w}\right)}^{2}+{\left(y_{w}^{\prime }-y_{w}\right)}^{2}+{\left(y_{w}^{\prime }-y_{w}\right)}^{2}}$$

**Figure 9:**
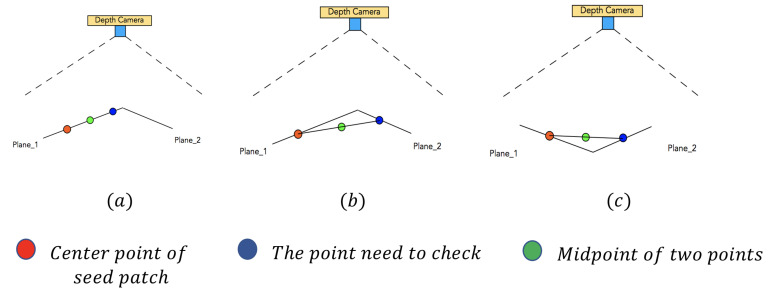
Cross section of overgrowing cases: (**a**) undergrowing; (**b**) overgrowing and convex to the camera; (**c**) overgrowing and concave to the camera.

As it shows in Figure 9, the cross-section of two connected planes ${\mathrm{plane}}_{1}$ and ${\mathrm{plane}}_{2}$ can be only concave or convex for the camera. We use a red point to represent the center of the seed patch; use a blue point to represent the current point $p_{c}$; and the green point is the midpoint between $p_{c}$ and the center point of the seed patch.

In case (a) of Figure 9, the current point $p_{c}$ is still undergrowing on ${\mathrm{plane}}_{1}$, so the green midpoint between $p_{c}$ and the center point of seed patch also locates on the ${\mathrm{plane}}_{1}$. Therefore, the offset error $error_{o}$ of the green midpoint calculated by Equation (19) will be very small.

In case (b) of Figure 9, the cross-section of ${\mathrm{plane}}_{1}$ and ${\mathrm{plane}}_{2}$ are convex to the camera. The current point $p_{c}$ has grown to ${\mathrm{plane}}_{2}$, and the midpoint is behind the two planes. In case (c) of Figure 9, the cross-section of ${\mathrm{plane}}_{1}$ and ${\mathrm{plane}}_{2}$ are concave to the camera. The current point $p_{c}$ also has grown to ${\mathrm{plane}}_{2}$, and the midpoint is above the two planes. In both cases (b) and case (c), the midpoint does not really exist in the depth image, so the $error_{o}$ in those cases will be much bigger than the one in case (a), which is not overgrowing. Through this approach, we can distinguish the overgrowing point easily in the iteration process and avoid overgrowing.

**Figure 10:**
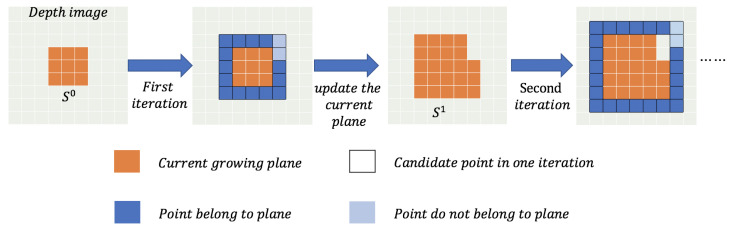
Iteration growing process from a 3 × 3 seed patch.

The iteration process of region growing is shown in Figure 10, and each iteration will process the points in a surrounding layer of the current plane, which is marked by the black border, and $S^{i}$ is the current plane after the *i*-th iteration. For instance, there are 16 points around the 3×3 seed patch that need to be processed in the first iteration of Figure 10. For each point $p_{c}$, we calculate the offset error $error_{o}$ of the midpoint between the current point and the center point of seed patch by Equation (19). Then, $error_{o}$ is compared to a threshold γ, which is set as γ=3 in our case, if $error_{o}<\gamma$, the current point belongs to the current plane.

After passing the threshold checking, we do the connecting checking by a connecting constraint: a point can be added to the current plane only when there is at least one of its 8-connected neighbors on the current growing plane. For example, in the second iteration in Figure 10, the two light blue points will not be accepted because there is no 8-connected neighbor of theirs on the current orange plane. If a pixel can pass all the checking, we add it to the current plane.

After each iteration, the current plane’s equation should be updated by the Linear Least Squares plane fitting approach. When the region can’t grow anymore, we can output the plane’s final equation, the range of plane on the *x*-axis $\left(min_{x},max_{x}\right)$ and the range of plane on the *y*-axis $\left(min_{y},max_{y}\right)$. The $\left(min_{x},max_{x}\right)$ and the $\left(min_{y},max_{y}\right)$ can roughly describe the range of plane. After processing each $\Theta_{c}$, we can get enough spatial information of each plane.

#### 3.2.4. Combine the Clusters on the Same Plane

Since some clusters may belong to the same plane and eventually grow to the same result, it is necessary to combine those clusters. In this section, a simplified graph clustering method is used for combining the clusters on the same plane to ensure that each cluster can represent a different plane.

Firstly, we build a simple unweighted graph, which is similar to the graph in Section 3.1, but the node is the $\Theta_{c}$ we have obtained, and all the edges have the same weight 1. Then, whether there is an edge between two nodes is decided by the similarity ξ between two planes. For example, there are two $\Theta_{c}$ with estimated equations $a_{1}x+b_{1}y-z+d_{1}=0$ and $a_{2}x+b_{2}y-z+d_{2}=0$, the center points’ coordinate of two clusters are $\left(x_{1},y_{1},z_{1}\right)$ and $\left(x_{2},y_{2},z_{2}\right)$. The similarity ξ of two planes is calculated by Equation (20):

(20) $$\xi =\left|a_{1}\times x_{2}+b_{1}\times y_{2}-z_{2}+d_{1}\right|+\left|a_{2}\times x_{1}+b_{2}\times y_{1}-z_{1}+d_{2}\right|$$

The ξ is not equal to zero even two clusters belong to same plane. Because a plane in the real world is always an approximate plane with slight deformation and there are small errors between the planes estimated by different seed patches. We calculate the ξ between each pair of the planes and compare to a threshold ι. The value of threshold ι depends on how much error between two planes we allow, and the relations between similarity ξ and error are shown in Table 4. In our system, we allow an error of about $5^{\circ }$, and the threshold is set to be ι=19, which works well around 20. For different requirements, the value of ι can be modified. If ξ<ι, those two nodes on the graph will be connected by an edge. After iterating through each pair of clusters, the Markov Cluster Algorithm introduced in Section 3.1 is applied to the graph. Finally, we can get some new clusters $\Theta_{set}$, which contains several $\Theta_{c}$.

To smooth the error, we use the average of all plane equations in the $\Theta_{set}$ to represent the plane’s equation of each $\Theta_{set}$. In addition, in each $\Theta_{set}$, the normal vector ${ñ}_{\mathrm{set}}$ is the average of all the $\Theta_{c}$’s normal vector ñ; the constant $d_{\mathrm{set}}$ is the average of all the $\Theta_{c}$’s constant d.

After the combination, each cluster $\Theta_{set}$ can represent a different plane from others. Moreover, to confirm our method’s effectiveness, we compared our region growing method with the region growing part of [57] in Section 4.2.2.

### 3.3. Sliding Checking Model

**Figure 11:**
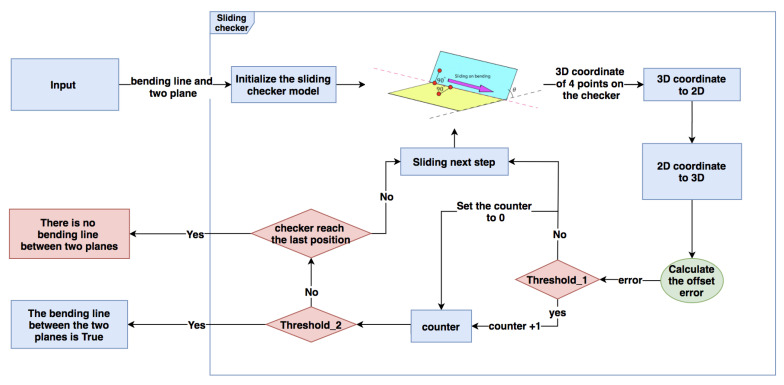
Apply Sliding checking model for bending line checking.

When we get each approximate plane’s equation on the deformed surface, it is necessary to check the adjacent relationship between each pair of planes. This section, which is in the green blocks of Figure 1, explains a sliding checking model for checking of the adjacent relationship. Since each plane we obtain in Section 3.2 comes from the local feature detected by the SIFT descriptor, it is difficult to know the adjacent relationship between the planes. However, through our novel sliding inspection model, this challenge can be effectively solved. Given the equations of two planes, the process shown in Figure 11 can be used to check whether two planes are neighbors, and there is a real bending line between two planes.

#### 3.3.1. Initializing Sliding Checking Model

**Figure 12:**
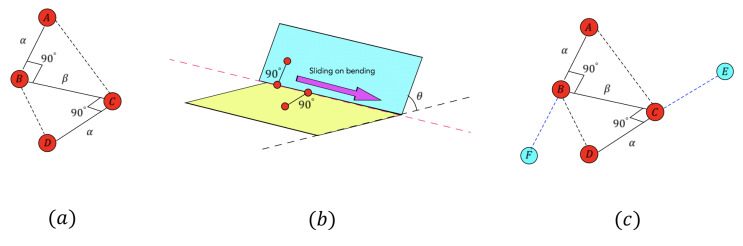
(**a**) Sliding checking model; (**b**) slide the model on the bending line; (**c**) the case of symmetry points.

Here, we will explain the configuration of the sliding checking model, which is shown in case (a) of Figure 12. It contains four points (A,B,C,D), where *B* and *C* are points on the calculated bending line with distance β, *A* is the point on one plane, and *C* is the point on another plane. Line segments AB and CD are perpendicular to BC with length AB=CD=α. To initialize the sliding checking model, in our case, we set α=5 and β=3.

In case (b) of Figure 12, we only need to change the position of point *B* to slide the model because the 3D coordinates of points (A,C,D) can be calculated from the coordinate of point *B*.

Due to the plane’s infinite ductility, while calculating the 3D coordinate of *A* and *D*, we will get two pairs of symmetry points (A,F) and (D,E). For example, there are three known conditions to calculate *A*: (1) The equation of the plane to which *A* belongs is given; (2)AB and BC are perpendicular to each other; (3) the length of AB=α. However, symmetry points *A* and *F* both meet three conditions, so we can get two coordinates, one of which is a point that does not really exist. The case (c) of Figure 12 shows two pairs of symmetry points (A,F) and (D,E), and *F* is the symmetry point of *A* with respect to line BC, and *E* is the symmetry point of *D* with respect to line BC. When we get four candidate pairs (A,D), (A,E), (F,D), and (F,E), only one pair can satisfy that two points exist, and it can be distinguished in the process of each sliding step.

**Figure 13:**
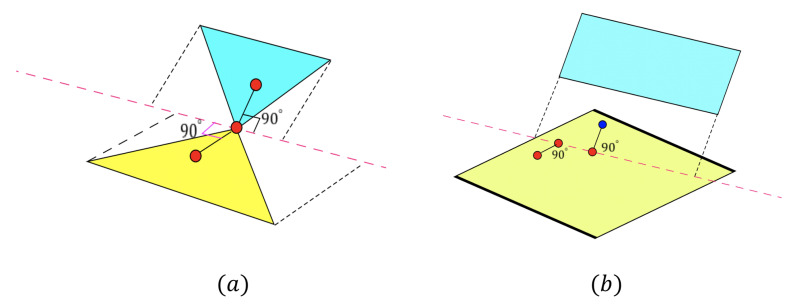
(**a**) Two planes only intersect at one point; (**b**) intersect line is on the expansion of one plane.

The reason for choosing four points rather than three to build the model is that three points are not enough to check the bending line in some special situations shown in Figure 13: In case (a), two planes only intersect at one point, and there is not a completed bending line. If we only use three points to check, all three points can pass the checking on the area around the intersect point. However, there is not a completed bending line between the planes. For the case (b), two planes do not contact each other, and the intersect line is on the expansion of the blue plane, so three red points really exist and can pass the checking with a small offset error $error_{o}$. However, the blue point located on the expansion of the blue plane does not really exist. It won’t pass the checking with a big $error_{o}$.

Therefore, only using the model with four points can ensure that there is a real bending line between two connected planes when all four points pass the checking with a small offset error $error_{o}$.

#### 3.3.2. Sliding the Model

After initializing the sliding model, the model will slide on the bending line step by step to check the existence of bending lines.

The start point and end point of the sliding are decided by the range of two planes obtained in Section 3.2. For each plane, we have obtained the boundary value on the *x*-axis $\left(min_{x},max_{x}\right)$ and *y*-axis $\left(min_{y},max_{y}\right)$. If the range of first plane is $\left(min_{x}1,max_{x}1\right)$ and $\left(min_{y}1,max_{y}1\right)$, the range of another one is $\left(min_{x}2,max_{x}2\right)$ and $\left(min_{y}2,max_{y}2\right)$, and the range of bending line must satisfy Equation (21), while $x_{b}$ is the *x*-value and $y_{b}$ is the *y*-value of point on the bending line:

(21) $$\begin{array}{c} x_{b}\in \left[min\left(min_{x}1,min_{x}2\right),max\left(max_{x}1,max_{x}2\right)\right]\\ y_{b}\in \left[min\left(min_{y}1,min_{y}2\right),max\left(max_{y}1,max_{y}2\right)\right] \end{array}$$

Then, we can start from the point on the bending line whose *x*-value is $min(min_{x}1,min_{x}2)$ and end at the point whose *x*-value is $max(max_{x}1,max_{x}2)$. This range is a little wider than the real length of the bending line, but only the point on the real bending can pass the offset checking.

In each sliding step, we first calculate the coordinate of point *C* and symmetry points (A,F) and (D,E). For each point with the calculated coordinate $\left(x_{w},y_{w},z_{w}\right)$, Equations (18) and (15) are used to get the coordinate $\left(x_{w}^{\prime },y_{w}^{\prime },z_{w}^{\prime }\right)$. Then, we calculate the offset error $error_{o}$ of each point and compare to a threshold λ=10. If the Pair(B,C) calculated by Equation (22) is True, then we will calculate the Pair of four candidates (A,D), (A,E), (F,D) and (F,E) and set a counter $C_{ij}$ for each of them. If one candidate Pair(i,j) satisfies the threshold λ=10, we add 1 to the $C_{ij}$, but, if the candidate Pair(i,j) doesn’t satisfy λ=10, the counter will be set to be $C_{ij}=0$. After operating the counters, we slide the model to next step and the size of a step is equal to β:

(22) $$Pair\left(p_{1},p_{2}\right)=\left(error_{o}\left(p_{1}\right)<\lambda \right)\wedge \left(error_{o}\left(p_{2}\right)<\lambda \right)$$

Only one of the candidates has two really existing points with low offset errors $error_{o}$, so the counter of it can be accumulated continuously. The bending line is considered true only when the maximum number of the counter is greater than half of the total number of steps we need to slide. By this kind of continuous checking, we can easily judge the existence of the bending line correctly, even though the threshold λ is a little rough.

Now that we know the adjacent relationship between each pair of planes. The performance of the entire system will be checked in Section 4.3.1.

## 4. Experimental Setup and Evaluation Results

We would like to confirm the operation of the proposed method and verify its effectiveness. Firstly, the experimental equipment and developmental environment used in our implementation are introduced in Section 4.1. Section 4.2 evaluates two techniques improved for understanding the deformed object based on existing technologies: (a) Clustering(Section 3.1), (b) Region Growing (Section 3.2). Then, we check the complete system’s performance by implementing our system on deformed papers and discuss the conditions for effective work in Section 4.3. Finally, a data structure is proposed to save the necessary information for the robot to reproduce the same deformation in Section 4.4.

### 4.1. Experimental Setup

In this section, we will introduce the experimental system settings for implementation. The two main devices of our system are one depth camera and one PC (personal computer).

The depth camera we chose is the Azure Kinect developed by Microsoft, and the specifications of Microsoft Azure Kinect are shown in Table 5.

Our approach uses the SIFT descriptor, which requires RGB images with high resolution, and the 4096x3072 RGB resolution of Azure Kinect is good enough to meet our needs. Regarding the accuracy of depth data, Azure Kinect is one of the best consumer depth cameras in recent years. It has good accuracy, and the measurable range is up to 5.46 m. The accuracy is good enough to estimate the deformation process, and the measurable range is acceptable for most of the robots to operate.

In order to run our system successfully, the minimum host PC hardware requirements are shown in Table 6. Because of the rapid development of computer hardware, most of the customer PC can meet our system’s hardware requirements.

The development environment of our system is shown in Table 7. The environment can be easily configured on a customer PC using the Linux operating system.

### 4.2. Evaluation of Improved Techniques

#### 4.2.1. Clustering

In order to evaluate the improvement of our weighted graph clustering method on the robustness, we compare our approach with the unweighted-graph method of [41] under different conditions such as different degrees of deformation, different objects, and occlusions. The unweighted graph method uses several fixed values to be the thresholds of constraints. When the conditions changed, the performance of this method can’t always be acceptable. However, our weighted graph approach can adapt to different conditions because each edge’s weight will change with conditions.

**Figure 14:**
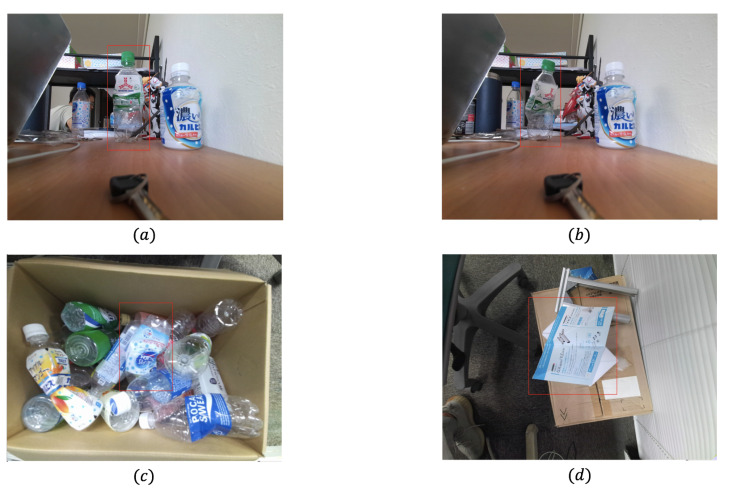
Four cases for clustering: (**a**) a bottle with low degree of deformation; (**b**) same bottle with case (**a**) but with a higher degree of deformation; (**c**) a deformed bottle in a box filled with different kinds of bottles; (**d**) a deformed paper with three bending lines.

We use four different cases in Figure 14. The objects in cases (a–c) are the deformed bottles, cases (a,b) are the same bottle, but the deformation degree of (b) is much larger than (a). In case (c), the deformed bottle is in a complex environment with occlusions. In case (d), the deformed object is a folded paper, and we can regard it as a combination of several approximate planes.

**Figure 15:**
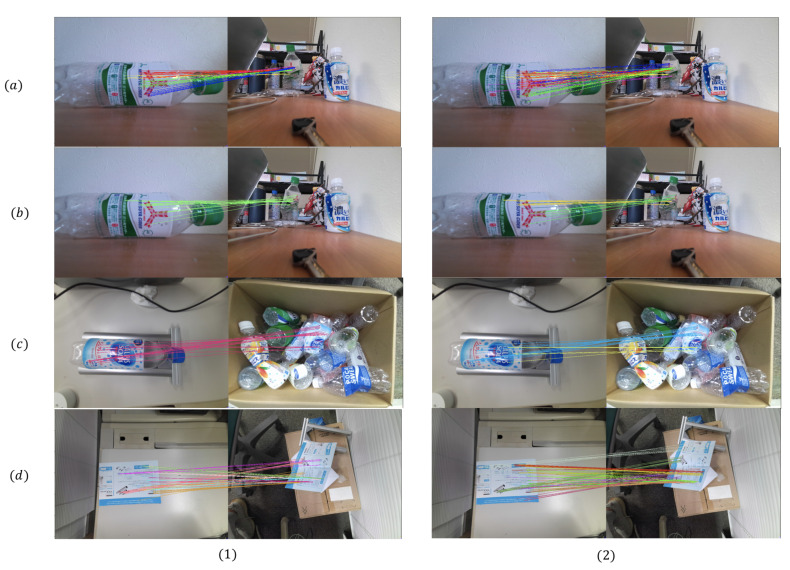
Result of clustering: (**1**) the unweighted graph clustering method of [41]; (**2**) our weighted graph clustering method; (**a**) bottle with low deformation; (**b**) bottle with high deformation; (**c**) bottle in complex environment; (**d**) deformed paper.

For each case, we use our weighted graph approach and the unweighted graph method of [41] to build two different graphs. Then, the Markov Cluster Algorithm introduced in Section 3.1 is applied to both graphs to get the clustering result. The result of clusters is shown in Figure 15 where each color represents a cluster. In cases (b,c), if the deformation degree is large or the object in a complex environment, we can get less SIFT feature points than standard cases. The unweighted graph method can’t work well under those challenging conditions. Some feature point matchings on the very different planes are divided into only one cluster. Our approach can still divide the feature point matchings into the right clusters in these extreme cases. In standard cases like (a,d), both methods can get several clusters on the different planes, but our approach can find more approximate planes even if the plane’s size is small or the feature is not much.

Therefore, our weighted graph approach has better robustness when the object is deformed with various folding angles or in a complex environment such as with occlusions. More potential planes can be detected by our approach under those challenging conditions.

#### 4.2.2. Region Growing Comparison

This section compares our new region growing method with the region growing part of [57] on different depth images to show the progress on solving the overgrowing problem. We just randomly set a seed patch on the depth image and apply two region growing processes with the same number of iterations.

**Figure 16:**
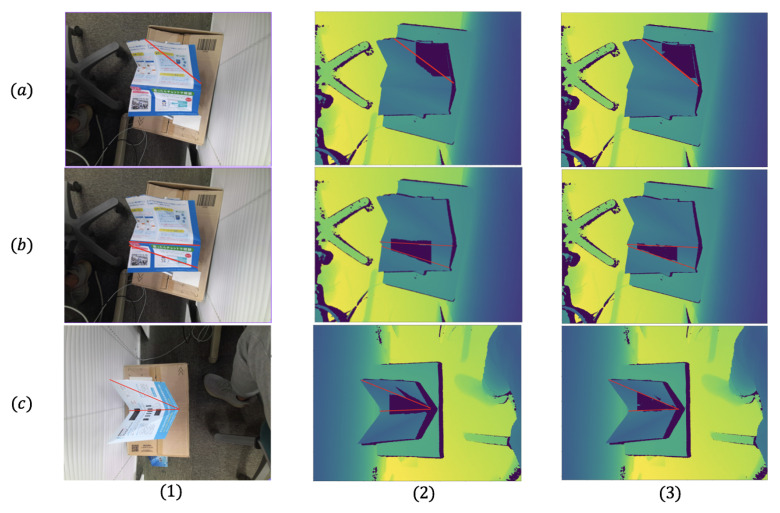
Three cases for region growing (The red line marks the bending line): (**1**) the RGB images of three cases; (**2**) the region growing method of [57]; (**3**) our approach; (**a,b**) two different planes on a same paper; (**c**) a plane on another different paper.

There are three cases (a–c) shown in Figure 16, cases (a,b) are on the same depth image but use different seed patches, case (c) is on the different depth images from (a,b). As we can see, the method of [57] in column (2), when the growing plane reaches the red bending line, doesn’t stop and still grows several iterations on the other plane. The overgrown area will cause a big error while estimating the equation of the plane. However, as it shows in column (3), our approach based on the offset error $error_{o}$ can definitely stop on the bending line.

Therefore, after the same number of iterations, our refined region growing approach can avoid overgrowing problems without any additional process. It can reduce the time cost in the calculation and improve the total system’s accuracy and speed.

### 4.3. Evaluation of Total Performance

#### 4.3.1. Total Performance

This section checks the performance of the entire system by testing our system on different deformed papers, whose process is the red part of Figure 1. The reason for choosing the paper to be the deformed object is that the paper’s patterns can provide enough features for the SIFT descriptor, and folding a piece of paper can keep each different part of the surface to be an approximate plane after the deformation.

We can obtain all the bending lines’ equations in the camera coordinate system by applying three core processes in Section 3 in order. Since the spatial information of bending lines is the present target of our system, all the errors during the performance of the whole process can be presented as the error of the bending lines. However, the ground truth of each bending line in the 3D coordinate system is hard to measure and get, but the bending line on the 2D RGB image can be manually labeled. Therefore, we calculate the projection on the 2D image of the 3D bending line and compare it with the ground truth to check the performance of our approach.

**Figure 17:**
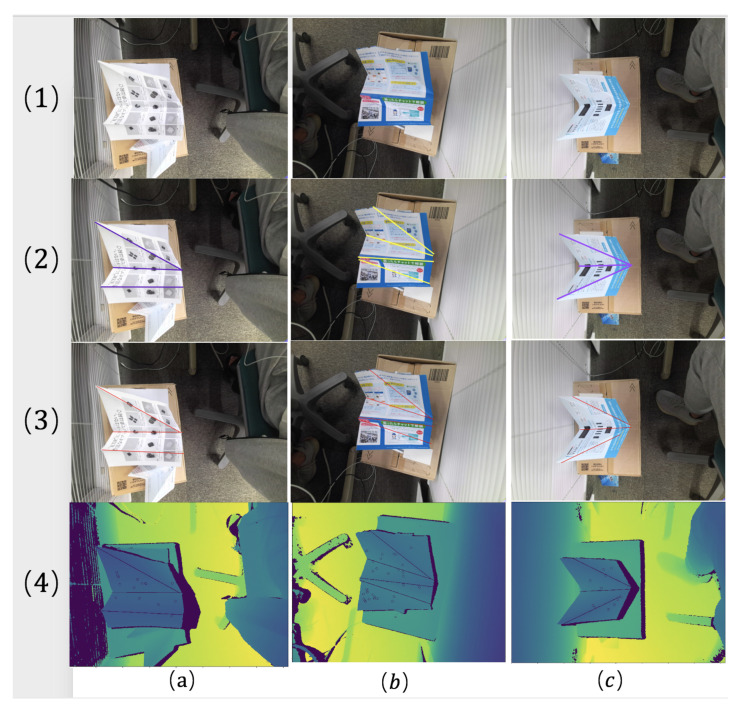
Three kinds of deformed paper (**a**–**c**): (**1**) RGB image; (**2**) ground truth of bending line; (**3**) projection of 3D bending line on the 2D RGB image; (**4**) projection of 3D bending line on the 2D depth image.

**Figure 18:**
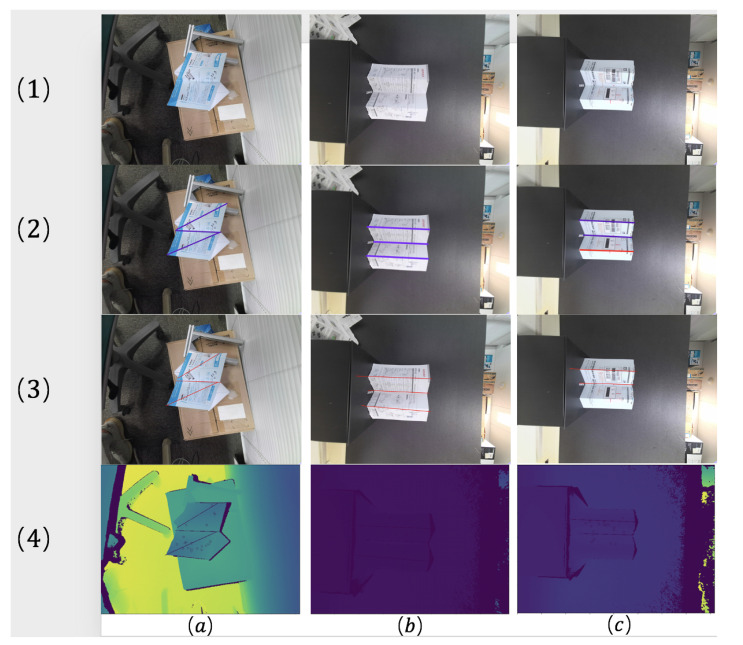
Three kinds of deformed paper (**a–c**): (**1**) RGB image; (**2**) ground truth of bending line; (**3**) projection of 3D bending line on the 2D RGB image; (**4**) projection of 3D bending line on 2D depth image.

Our system is tested on six different deformed papers, which are shown in Figure 17a–c and Figure 18a–c. The first row (1) shows the deformed paper’s RGB image, and the ground truth of the bending lines is manually labeled in the second row (2). In the third row (3) and fourth row (4), we calculate each bending line’s projection on the 2D image and draw the line on the RGB image and depth image.

As we can see, in the cases Figure 17a,b and Figure 18a, the bending lines we have drawn are very close to the ground truth. For the cases Figure 17c and Figure 18b, there are small offsets between the bending lines we calculated and the ground truth. This kind of offset is mainly caused by the slight deformation on some planes of paper. Due to the soft material properties of paper, these planes are approximate planes, so when we use plane equations to represent approximate planes, and there must be some errors. In case Figure 18c, there are three bending lines in the ground truth, but only two bending lines are detected in our result. The reason for losing one bending line is that there are not enough SIFT features detected from one of the planes. If some planes don’t have enough patterns for the SIFT descriptor, it is difficult for them be detected and some bending lines can be lost.

To obtain a quantitative evaluation, we calculate the angle between the 2D projection of our result and the 2D manually labeled ground truth as the error. For all six cases, Table 8 shows the average angular error on each bending line in each case, and the total mean error is around $1.59^{\circ }$. About the conditions such as the position relationship between object and camera, they are similar in cases (e,f), and they are different in other cases, but the average angular errors are similar in all those cases, less than $3^{\circ }$. Therefore, we think that the angular error is less than $3^{\circ }$ has a certain degree of generality.

The required accuracy of the reproduction task by a robot depends on the size and complexity of the target object and the quality of work required. One research work on the human eyes shows that the smallest angular difference for which correct discriminations could be made for a reasonable range of stimulus sizes and eccentricities is around 1.29–1.83${}^{\circ }$[58], which is similar to our total mean error $1.59^{\circ }$. Therefore, we think that a robot can perform some reproduction operations based on data with this kind of accuracy.

#### 4.3.2. Discussion of Working Conditions

After checking the entire system’s performance by good or bad cases, we would like to discuss our system’s limitations and effective work conditions in this section.

**Figure 19:**
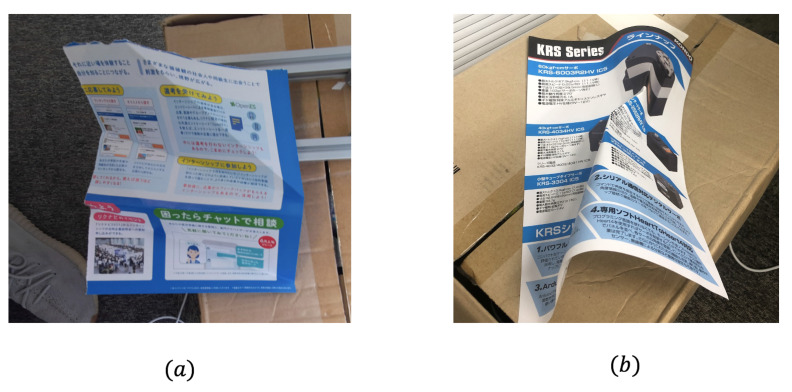
Deformed paper: (**a**) Only by folding; (**b**) Irregularly deformed.

**(1) Style of deformation:** Our approach now can work well with the deformation caused by folding like (a) in Figure 19 in which each segmental element can be regarded as an approximate plane. However, for irregular deformation like (b) in Figure 19, the process of deformation is hard to be described by simple plane equations.

Another limitation is whether we can get enough SIFT features on each plane. If we can’t get a SIFT key points cluster on one plane, this plane will be missed, and the complete deformation process can’t be obtained. Two factors can affect the feature getting: the paper’s patterns and the relative position between the camera and the object.

**(2) Pattern of surface:** The pattern and texture of the surface are important to the SIFT descriptor, so our system can’t handle the object such as a blank paper or a poorly colored paper. To verify the influence of the pattern on the surface, cases (e,f) in Figure 18 use the papers in the same size and deformed into the same shape; only the patterns of each paper are different. The patterns of case (f) are concentrated in the middle area and the edge area is almost blank, so one segmental element with the little pattern is not detected. The region growing process is only applied to the plane with a feature point cluster on it. If there are not enough SIFT features on a plane, we will miss them. Moreover, the missing plane will make the bending lines around it also be missed, like case (f).

Therefore, there are enough patterns on most of the papers. The relative position between the camera and the plane also influences the SIFT feature point matching, which can also be divided into two main factors: the distance from the camera and the plane’s pose.

**Figure 20:**
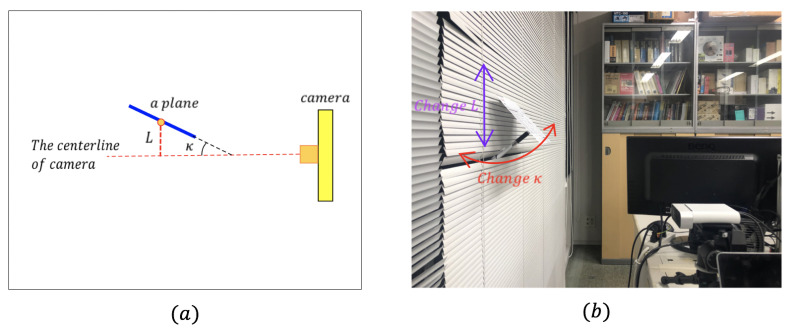
Distance and angle to object: (**a**) Schematic diagram. (**b**) Configuration change of the κ and *L*.

**(3) Distance and angle to object:** About the distance, for the high 4096×3072 RGB resolution of Azure Kinect. The result of SIFT feature matching is acceptable within a 1-m distance. Therefore, we mainly consider the influence of the plane pose. As shown in (a) of Figure 20, the pose of a plane which will influence the feature detection can be divided into two main factors: the angle between the plane and the centerline of camera κ, the distance between the center point of plane, and the centerline of camera *L*.

To show the influence of κ and *L*, we use a simple configuration shown in Figure 20b, which can change the κ and *L* separately. Then, we use a piece of paper to represent a plane. Every time, we only change κ or *L* and keep other conditions the same. Moreover, we draw a circle around each cluster’s center on depth images to show feature detection performance.

**Figure 21:**
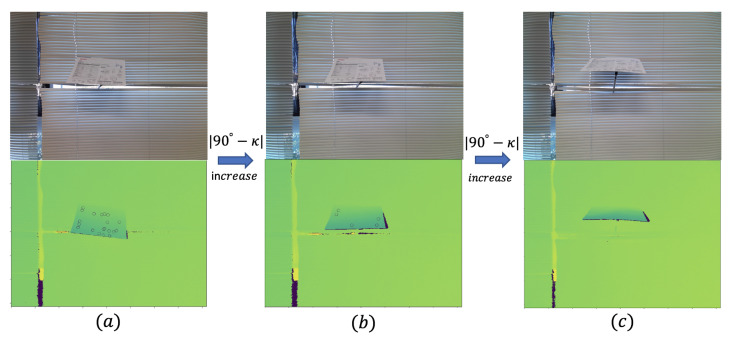
Change in tilt angle: increasing $|90^{\circ }-\kappa |$ as (a)→(b)→(c).

In Figure 21, we only change the κ and keep other factors to be the same. The best condition is when the $\kappa =90^{\circ }$, which means that the camera’s centerline is perpendicular to the plane. When $|90^{\circ }-\kappa |$ increases, we can see cases (a–c) in Figure 21, and the number of clusters we can obtain will decrease.

**Figure 22:**
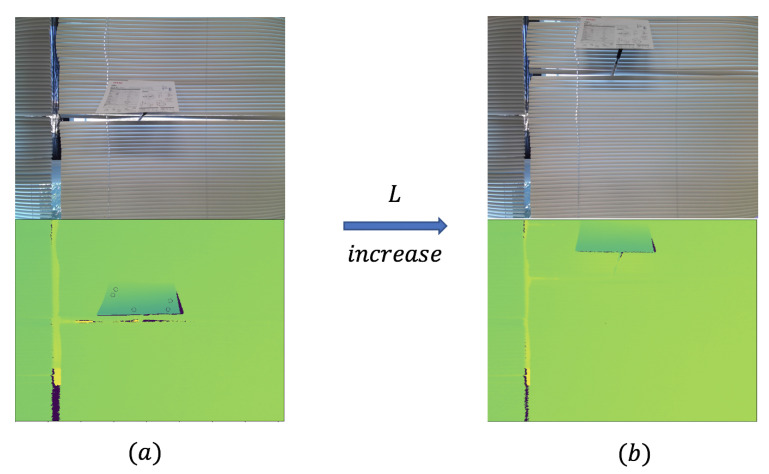
Displacement from center as increasing *L*: (a)→(b).

In Figure 22, we change the *L* and don’t change other conditions. Furthermore, the best condition is when the L=0, which means the center of the plane is on the camera’s centerline. When the *L* increases, we can see the cases (a,b) in Figure 22, and the features detected decrease.

Therefore, our method requires that the $|90^{\circ }-\kappa |$ and *L* should not be too large for a plane, or if we are not able to detect enough SIFT feature points from the plane, the plane will be missed and won’t participate in subsequent processing like region growing process and sliding checking process. However, if there is at least one cluster generated on each plane, our system can get an acceptable final result for deformation analysis.

In summary, the proposed system can have good performance with around $1.59^{\circ }$ average angular error when two conditions are met: (1) The deformation is only caused by folding, which means each segmental element of the deformed surface can be regarded as an approximate plane. (2) Each potential plane can be detected by the non-rigid point matching method from the RGB image, which means that the surface can provide enough features for SIFT descriptor and there is at least one cluster $\Theta_{c}$ generated on each plane; for example, the object with the following surface texture can’t be supported: (a) monochromatic and no pattern, (b) the same pattern is repeated, and (c) the pattern has little change.

### 4.4. Data for Reproduction by a Robot

After verifying the accuracy of our proposed system, we would like to generate the input data for a robot with human-like dexterous hands and fingers to produce the same deformation in the future. Data must include at least (1) equation (position and orientation) of the upward bending line and the downward bending lines on the folded paper and the (2) relationship (whether adjacent or not, and angle) between folded planes.

**Figure 23:**
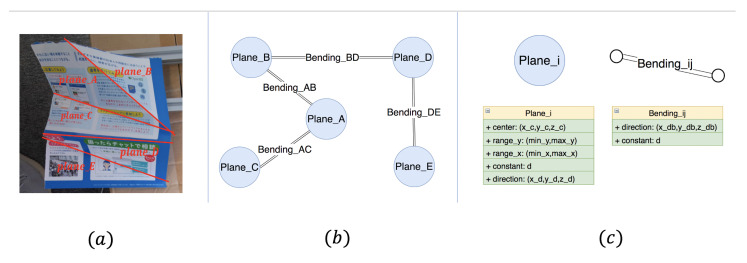
Data for reproduction: (**a**) Folded paper with four bending lines and five planes; (**b**) generated $Graph_{r}$; (**c**) node-edge information.

Our input for the robot is a graph-like data structure $Graph_{r}$ shown in Figure 23b. In the $Graph_{r}$, each node represents a plane, and each edge between two nodes represents a bending line between two planes. Therefore, for each plane we get in Section 3.2, we will create a node in the graph, which saves the center point of the cluster, the equation, and the approximate range of the plane. For each real bending line we get in Section 3.3, we use an edge to connect two nodes to which the bending line belongs, and each edge saves the equation of the bending line and the folding angle between two planes. All of the equations are in the camera coordinate system.

For each node, we can find the same corresponding area on another similar object, which is in the initial state. It is possible to make a robot reproduce the same deformation of another similar object.

These kinds of data help to understand the deformation process, which is essential for the robot to complete the folding task step by step. The reproducing deformation process in the future can be like that: first, we find an edge in the $Graph_{r}$, and, for the two nodes connected by this edge, we can find two corresponding areas in another similar paper, which is in the initial state. Then, a robot can operate those two corresponding areas to generate a bending line, which is the same as the one saved in the edge.

If the functions of the robot are powerful enough with human-like dexterous hands and fingers in the future, after dealing with each edge in the $Graph_{r}$ in a certain order, it is possible to reproduce the same deformation as the one in the given image.

## 5. Conclusions

This paper has introduced a system that can estimate the deformation process of a deformed flat object (folded plane) and generate the input data for a robot with human-like dexterous hands and fingers to reproduce the same deformation of another similar object. The system is based on processing the RGB data and depth data with three core techniques: (1) A weighted graph clustering method for non-rigid point matching and clustering. It can find the potential planes and establish the correspondence between each pair of planes on two RGB images. (2) A refined region growing method for plane detection on depth data based on an offset error defined by ourselves. It can avoid the overgrowing problem without any additional process to reduce the time cost on calculation. (3) A novel sliding checking model to check the bending line and adjacent relationship between each pair of planes. With the information about each segmental element, it would be possible to estimate the deformation process.

Through several evaluation experiments, we have evaluated the improvement of two core techniques and the entire system’s performance in Section 4:

(a) The comparison of clustering in Section 4.2.1 shows that our weighted graph approach has better robustness than the old method of [41] when the object is deformed with various folding angles or in a complex environment such as with occlusions.
(b) The experiment of region growing in Section 4.2.2 shows that our refined region growing approach can avoid overgrowing without any additional process. It can reduce the time cost in calculation and improve the accuracy and speed of the total system.
(c) In Section 4.3.1, we have evaluated the total performance and confirmed that its performance with around $1.59^{\circ }$ average angular error is acceptable based on which a robot would be able to reproduce the same deformation by folding the paper.
(d) Section 4.3.2 has discussed the conditions for the entire system to work effectively. Our system can have good performance when the following two conditions are met: (1) Deformation is only caused by folding. (2) Each potential plane can be detected by the non-rigid point matching method from the RGB image. In addition, the object with the following surface texture can’t be supported: (a) monochromatic and no pattern, (b) the same pattern is repeated, and (c) the pattern has little change.

In summary, for the deformation of an object caused by folding, if our system can get at least one feature point cluster on each plane, it can get the spatial information of each bending line and flat plane with acceptable accuracy. With this generated information, it is possible to reproduce the same deformation on another similar object by a robot in the future.

About the future works, the subject of this paper is a folded plane, but we will develop it into a robotic reproduction of general object deformation. In order to deal with the irregular deformation, one possible way is dividing each irregular plane into a set of meshes with neighborhood relations, and some deep learning technologies may be helpful to solve the calculation time problem. In order to solve the limitation of SIFT feature detection, multiple sensors can be used to avoid no blind spots, and deep learning techniques can improve the performance of finding potential planes.

## Figures and Tables

**Table 1:** Comparative overview.

Item	Priori Knowledge Based (RGB)	NRSFM (RGB)	Template-Based (RGB-D)	Templateless (RGB-D)	Our Approach (RGB-D)
Input data	RGB images	RGB images	RGB images with depth data	RGB images with depth data	RGB images with depth data
Priori knowledge	Models or reference point correspondence	Doesn’t require	Pre-defined template	Doesn’t require	Doesn’t require
Accuracy	Acceptable	Acceptable	Higher than RGB methods	Higher than RGB methods	Higher than RGB methods
Output	Global result in virtual space	Global result in virtual space	Global result in real space	Global result in real space	Segmental elements with their relationship in real space
For robot understanding	Hard	Hard	Hard	Hard	Easy

**Table 2:** Comparative overview.

Item	Outlier Removal by Constraints	Iteration Based	Previous Graph-Based	Our Approach
Robustness	Sensitive to outliers	Sensitive to outliers	Sensitive to changing conditions	Robust with changing conditions
Local neighborhood structures	can’t preserve	can’t preserve	can preserve	can preserve

**Table 3:** Comparative overview.

Item	RANSAC	Hough Transform	Previous Region Growing	Our Approach
Accuracy	High to large planes	High	High	High
Computational load	High	Very high	High	High
Robustness	Weak to complex planar structures	Robust to noisy environment	Weak to the overgrowing	Avoid overgrowing problem

**Table 4:** Relations between ξ and error.

Error between Two Planes	Similarity ξ
Around $5^{\circ }$	Around 20
Around $10^{\circ }$	Around 30
Around $20^{\circ }$	Around 50
Around $30^{\circ }$	Around 80

**Table 5:** Specifications of Microsoft Azure Kinect.

Item	Specification
Product	Microsoft Azure Kinect
Dimensions	103 × 39 × 126 mm
Weight	440 g
Interface	USB 2.0/3.0
Max Resolution	1024× 1024(depth)/4096× 3072(RGB)
Depth measurable range	0.25–5.46 m
Typical systematic error	<11 mm + 0.1% of distance without multi-path interference
Tandom error	≤17 mm
FPS (Frames-per-second)	0, 5, 15, 30 FPS
Power consumption	5.9 W
SDK system requirements	Windows 10 April 2018 (Version 1803, OS Build 17134) release (×64)Linux Ubuntu 18.04 (×64), with a GPU driver that uses OpenGLv4.4

**Table 6:** Minimum host PC hardware requirements.

Item	Specification
CPU/GPU	Seventh Gen Intel^®^ CoreTM i3 Processor (Dual Core 2.4 GHz with HD620 GPU or faster)
Memory	4 GB Memory
Interface	Dedicated USB3 port
Graphics driver	Graphics driver support for OpenGL 4.4 or DirectX 11.0

**Table 7:** Development environment.

Item	Specification
Operating system	Linux Ubuntu 18.04 (×64)
Programming language	Python 3.6 or later version
Libraries and Packages	Azure Kinect sensor SDKopencv-python, matplotlib pyk4a, numpy, tkinter
Programming IDE	PyCharm

**Table 8:** Angular error to the 2D ground truth.

Case	Average Angular Error
(a)	$1.67^{\circ }$
(b)	$2.00^{\circ }$
(c)	$2.67^{\circ }$
(d)	$1.67^{\circ }$
(e)	$1.00^{\circ }$
(f)	$0.50^{\circ }$
Average	$1.59^{\circ }$

## Data Availability

The data presented in this study are available on request from the corresponding author.

## References

[1] Cohen L.D., Cohen I. (1993). Finite-element methods for active contour models and balloons for 2-D and 3-D images. IEEE Trans. Pattern Anal. Mach. Intell..

[2] Metaxas D., Terzopoulos D. (1993). Shape and nonrigid motion estimation through physics-based synthesis. IEEE Trans. Pattern Anal. Mach. Intell..

[3] Urtasun R., Fleet D.J., Hertzmann A., Fua P. Priors for people tracking from small training sets. Proceedings of the Tenth IEEE International Conference on Computer Vision (ICCV’05).

[4] Perriollat M., Bartoli A. A Quasi-Minimal Model for Paper-Like Surfaces. Proceedings of the 2007 IEEE Conference on Computer Vision and Pattern Recognition.

[5] McInerney T., Terzopoulos D. A finite element model for 3D shape reconstruction and nonrigid motion tracking. Proceedings of the 1993 (4th) International Conference on Computer Vision.

[6] Malti A., Bartoli A., Collins T. A pixel-based approach to template-based monocular 3D reconstruction of deformable surfaces. Proceedings of the 2011 IEEE International Conference on Computer Vision Workshops (ICCV Workshops).

[7] Bregler C., Hertzmann A., Biermann H. Recovering non-rigid 3D shape from image streams. Proceedings of the IEEE Conference on Computer Vision and Pattern Recognition. CVPR 2000 (Cat. No.PR00662).

[8] Torresani L., Hertzmann A., Bregler C. (2008). Nonrigid Structure-from-Motion: Estimating Shape and Motion with Hierarchical Priors. IEEE Trans. Pattern Anal. Mach. Intell..

[9] Akhter I., Sheikh Y., Khan S. In defense of orthonormality constraints for nonrigid structure from motion. Proceedings of the 2009 IEEE Conference on Computer Vision and Pattern Recognition.

[10] Gotardo P.F.U., Martinez A.M. Kernel non-rigid structure from motion. Proceedings of the 2011 International Conference on Computer Vision.

[11] Varol A., Salzmann M., Tola E., Fua P. Template-free monocular reconstruction of deformable surfaces. Proceedings of the 2009 IEEE 12th International Conference on Computer Vision.

[12] Taylor J., Jepson A.D., Kutulakos K.N. Non-rigid structure from locally-rigid motion. Proceedings of the 2010 IEEE Computer Society Conference on Computer Vision and Pattern Recognition.

[13] Russell C., Fayad J., Agapito L. (2011). Energy based multiple model fitting for non-rigid structure from motion. CVPR.

[14] Salzmann M., Hartley R., Fua P. Convex Optimization for Deformable Surface 3-D Tracking. Proceedings of the 2007 IEEE 11th International Conference on Computer Vision.

[15] Wang C., Li X., Liu Y. Monocular 3D Tracking of Deformable Surfaces Using Linear Programming. Proceedings of the 2010 20th International Conference on Pattern Recognition.

[16] Tsoli A., Argyros A.A. Patch-Based Reconstruction of a Textureless Deformable 3D Surface from a Single RGB Image. Proceedings of the 2019 IEEE/CVF International Conference on Computer Vision Workshop (ICCVW).

[17] Bednarik J., Fua P., Salzmann M. Learning to Reconstruct Texture-Less Deformable Surfaces from a Single View. Proceedings of the 2018 International Conference on 3D Vision (3DV).

[18] Ye G., Liu Y., Hasler N., Ji X., Dai Q., Theobalt C. (2012). Performance capture of interacting characters with handheld kinects. Proceedings of the European Conference on Computer Vision.

[19] Elanattil S., Moghadam P., Denman S., Sridharan S., Fookes C. Skeleton Driven Non-Rigid Motion Tracking and 3D Reconstruction. Proceedings of the 2018 Digital Image Computing: Techniques and Applications (DICTA).

[20] Lim J., Yang M.-H. A direct method for modeling non-rigid motion with thin plate spline. Proceedings of the 2005 IEEE Computer Society Conference on Computer Vision and Pattern Recognition (CVPR’05).

[21] Kozlov C., Slavcheva M., Ilic S. Patch-Based Non-rigid 3D Reconstruction from a Single Depth Stream. Proceedings of the 2018 International Conference on 3D Vision (3DV).

[22] Tsoli A., Argyros A.A. Tracking Deformable Surfaces That Undergo Topological Changes Using an RGB-D Camera. Proceedings of the 2016 Fourth International Conference on 3D Vision (3DV).

[23] Paulus C.J., Haouchine N., Cazier D., Cotin S. Augmented Reality during Cutting and Tearing of Deformable Objects. Proceedings of the 2015 IEEE International Symposium on Mixed and Augmented Reality.

[24] Zhang Q., Fu B., Ye M., Yang R. Quality Dynamic Human Body Modeling Using a Single Low-cost Depth Camera. Proceedings of the IEEE Conference on Computer Vision and Pattern Recognition (CVPR).

[25] Dou M., Taylor J., Fuchs H., Fitzgibbon A., Izadi S. 3D scanning deformable objects with a single RGBD sensor. Proceedings of the 2015 IEEE Conference on Computer Vision and Pattern Recognition (CVPR).

[26] Wang K., Zhang G., Xia S. (2017). Templateless Non-Rigid Reconstruction and Motion Tracking With a Single RGB-D Camera. IEEE Trans. Image Process..

[27] Slavcheva M., Baust M., Cremers D., Ilic S. KillingFusion: Non-rigid 3D Reconstruction without Correspondences. Proceedings of the 2017 IEEE Conference on Computer Vision and Pattern Recognition (CVPR).

[28] Yang J., Guo D., Li K., Wu Z., Lai Y. (2019). Global 3D Non-Rigid Registration of Deformable Objects Using a Single RGB-D Camera. IEEE Trans. Image Process..

[29] Cui C., Ngan K.N. (2013). Global Propagation of Affine Invariant Features for Robust Matching. IEEE Trans. Image Process..

[30] Chum O., Matas J. Matching with PROSAC - progressive sample consensus. Proceedings of the 2005 IEEE Computer Society Conference on Computer Vision and Pattern Recognition (CVPR’05).

[31] Zhu J., Lyu M.R. Progressive Finite Newton Approach To Real-time Nonrigid Surface Detection. Proceedings of the 2007 IEEE Conference on Computer Vision and Pattern Recognition.

[32] Lowe D.G. Object recognition from local scale-invariant features. Proceedings of the Seventh IEEE International Conference on Computer Vision.

[33] Ma J., Zhao J., Tian J., Yuille A.L., Tu Z. (2014). Robust Point Matching via Vector Field Consensus. IEEE Trans. Image Process..

[34] Besl P.J., McKay N.D. (1992). A method for registration of 3-D shapes. IEEE Trans. Pattern Anal. Mach. Intell..

[35] Chui H., Rangarajan A. A new algorithm for non-rigid point matching. Proceedings of the IEEE Conference on Computer Vision and Pattern Recognition. CVPR 2000 (Cat. No.PR00662).

[36] Sofka M., Yang G., Stewart C.V. Simultaneous Covariance Driven Correspondence (CDC) and Transformation Estimation in the Expectation Maximization Framework. Proceedings of the 2007 IEEE Conference on Computer Vision and Pattern Recognition.

[37] Zheng Y., Doermann D. (2006). Robust point matching for nonrigid shapes by preserving local neighborhood structures. IEEE Trans. Pattern Anal. Mach. Intell..

[38] Izadi M., Saeedi P. (2012). Robust Weighted Graph Transformation Matching for Rigid and Nonrigid Image Registration. IEEE Trans. Image Process..

[39] Leordeanu M., Hebert M. A spectral technique for correspondence problems using pairwise constraints. Proceedings of the Tenth IEEE International Conference on Computer Vision (ICCV’05).

[40] Qu H., Wang J., Li B., Yu M. (2017). Probabilistic Model for Robust Affine and Non-Rigid Point Set Matching. IEEE Trans. Pattern Anal. Mach. Intell..

[41] Tarashima S., Shimamura J., Kinebuchi T., Satah S. Keypoint Matching for Non-Rigid Object via Locally Consistent Visual Pattern Mining. Proceedings of the 2018 25th IEEE International Conference on Image Processing (ICIP).

[42] Macropol K. (2009). Clustering on Graphs: The Markov Cluster Algorithm (MCL).

[43] Fischler M.A., Bolles R.C. (1981). Random sample consensus: A paradigm for model fitting with applications to image analysis and automated cartography. Commun. ACM.

[44] Qian X., Ye C. (2014). NCC-RANSAC: A Fast Plane Extraction Method for 3-D Range Data Segmentation. IEEE Trans. Cybern..

[45] Yue W., Lu J., Zhou W., Miao Y. A new plane segmentation method of point cloud based on mean shift and RANSAC. Proceedings of the 2018 Chinese Control And Decision Conference (CCDC).

[46] Xu D., Li F., Wei H. 3D Point Cloud Plane Segmentation Method Based on RANSAC And Support Vector Machine. Proceedings of the 2019 14th IEEE Conference on Industrial Electronics and Applications (ICIEA).

[47] Hulik R., Spanel M., Smrz P., Materna Z. (2014). Continuous plane detection in point-cloud data based on 3D Hough Transform. J. Vis. Commun. Image Represent..

[48] Kiryati N., Eldar Y., Bruckstein A.M. (1991). A probabilistic Hough transform. Pattern Recognit..

[49] Huang H., Brenner C. Rule-based roof plane detection and segmentation from laser point clouds. Proceedings of the 2011 Joint Urban Remote Sensing Event.

[50] Fernandes L.A., Oliveira M.M. (2008). Real-time line detection through an improved Hough transform voting scheme. Pattern Recognit..

[51] Hähnel D., Burgard W., Thrun S. (2003). Learning compact 3D models of indoor and outdoor environments with a mobile robot. Robot. Auton. Syst..

[52] Poppinga J., Vaskevicius N., Birk A., Pathak K. Fast plane detection and polygonalization in noisy 3D range images. Proceedings of the 2008 IEEE/RSJ International Conference on Intelligent Robots and Systems.

[53] Xiao J., Adler B., Zhang H. 3D point cloud registration based on planar surfaces. Proceedings of the 2012 IEEE International Conference on Multisensor Fusion and Integration for Intelligent Systems (MFI).

[54] Smisek J., Jancosek M., Pajdla T. (2013). 3D with Kinect. Consumer Depth Cameras for Computer Vision.

[55] Deng Z., Todorovic S., Latecki L.J. (2017). Unsupervised object region proposals for RGB-D indoor scenes. Comput. Vis. Image Underst..

[56] Holz D., Behnke S. (2014). Approximate triangulation and region growing for efficient segmentation and smoothing of range images. Robot. Auton. Syst..

[57] Jin Z., Tillo T., Zou W., Zhao Y., Li X. (2019). Robust Plane Detection Using Depth Information From a Consumer Depth Camera. IEEE Trans. Circuits Syst. Video Technol..

[58] Sally S.L., Gurnsey R. (2003). Orientation discrimination in foveal and extra-foveal vision: Effects of stimulus bandwidth and contrast. Vis. Res..

